# Evolution of Extensively Drug-Resistant Tuberculosis over Four Decades: Whole Genome Sequencing and Dating Analysis of *Mycobacterium tuberculosis* Isolates from KwaZulu-Natal

**DOI:** 10.1371/journal.pmed.1001880

**Published:** 2015-09-29

**Authors:** Keira A. Cohen, Thomas Abeel, Abigail Manson McGuire, Christopher A. Desjardins, Vanisha Munsamy, Terrance P. Shea, Bruce J. Walker, Nonkqubela Bantubani, Deepak V. Almeida, Lucia Alvarado, Sinéad B. Chapman, Nomonde R. Mvelase, Eamon Y. Duffy, Michael G. Fitzgerald, Pamla Govender, Sharvari Gujja, Susanna Hamilton, Clinton Howarth, Jeffrey D. Larimer, Kashmeel Maharaj, Matthew D. Pearson, Margaret E. Priest, Qiandong Zeng, Nesri Padayatchi, Jacques Grosset, Sarah K. Young, Jennifer Wortman, Koleka P. Mlisana, Max R. O'Donnell, Bruce W. Birren, William R. Bishai, Alexander S. Pym, Ashlee M. Earl

**Affiliations:** 1 Division of Pulmonary and Critical Care Medicine, Brigham and Women’s Hospital, Harvard Medical School, Boston, Massachusetts, United States of America; 2 KwaZulu-Natal Research Institute for TB and HIV (K-RITH), Durban, South Africa; 3 Broad Institute of MIT and Harvard, Cambridge, Massachusetts, United States of America; 4 Delft Bioinformatics Lab, Delft University of Technology, Delft, The Netherlands; 5 Medical Research Council, Durban, South Africa; 6 Center for Tuberculosis Research, Johns Hopkins School of Medicine, Baltimore, Maryland, United States of America; 7 School of Laboratory Medicine and Medical Sciences, University of KwaZulu-Natal, Durban, South Africa; 8 National Health Laboratory Service, Durban, South Africa; 9 Centre for the AIDS Programme of Research in South Africa (CAPRISA), Durban, South Africa; 10 Division of Pulmonary, Allergy, and Critical Care Medicine, Columbia University College of Physicians and Surgeons, New York, United States of America; 11 Department of Epidemiology, Columbia Mailman School of Public Health, New York, United States of America; University of California, San Francisco, UNITED STATES

## Abstract

**Background:**

The continued advance of antibiotic resistance threatens the treatment and control of many infectious diseases. This is exemplified by the largest global outbreak of extensively drug-resistant (XDR) tuberculosis (TB) identified in Tugela Ferry, KwaZulu-Natal, South Africa, in 2005 that continues today. It is unclear whether the emergence of XDR-TB in KwaZulu-Natal was due to recent inadequacies in TB control in conjunction with HIV or other factors. Understanding the origins of drug resistance in this fatal outbreak of XDR will inform the control and prevention of drug-resistant TB in other settings. In this study, we used whole genome sequencing and dating analysis to determine if XDR-TB had emerged recently or had ancient antecedents.

**Methods and Findings:**

We performed whole genome sequencing and drug susceptibility testing on 337 clinical isolates of *Mycobacterium tuberculosis* collected in KwaZulu-Natal from 2008 to 2013, in addition to three historical isolates, collected from patients in the same province and including an isolate from the 2005 Tugela Ferry XDR outbreak, a multidrug-resistant (MDR) isolate from 1994, and a pansusceptible isolate from 1995. We utilized an array of whole genome comparative techniques to assess the relatedness among strains, to establish the order of acquisition of drug resistance mutations, including the timing of acquisitions leading to XDR-TB in the LAM4 spoligotype, and to calculate the number of independent evolutionary emergences of MDR and XDR. Our sequencing and analysis revealed a 50-member clone of XDR *M*. *tuberculosis* that was highly related to the Tugela Ferry XDR outbreak strain. We estimated that mutations conferring isoniazid and streptomycin resistance in this clone were acquired 50 y prior to the Tugela Ferry outbreak (*katG* S315T [isoniazid]; *gidB* 130 bp deletion [streptomycin]; 1957 [95% highest posterior density (HPD): 1937–1971]), with the subsequent emergence of MDR and XDR occurring 20 y (*rpoB* L452P [rifampicin]; *pncA* 1 bp insertion [pyrazinamide]; 1984 [95% HPD: 1974–1992]) and 10 y (*rpoB* D435G [rifampicin]; *rrs* 1400 [kanamycin]; *gyrA* A90V [ofloxacin]; 1995 [95% HPD: 1988–1999]) prior to the outbreak, respectively. We observed frequent de novo evolution of MDR and XDR, with 56 and nine independent evolutionary events, respectively. Isoniazid resistance evolved before rifampicin resistance 46 times, whereas rifampicin resistance evolved prior to isoniazid only twice. We identified additional putative compensatory mutations to rifampicin in this dataset. One major limitation of this study is that the conclusions with respect to ordering and timing of acquisition of mutations may not represent universal patterns of drug resistance emergence in other areas of the globe.

**Conclusions:**

In the first whole genome-based analysis of the emergence of drug resistance among clinical isolates of *M*. *tuberculosis*, we show that the ancestral precursor of the LAM4 XDR outbreak strain in Tugela Ferry gained mutations to first-line drugs at the beginning of the antibiotic era. Subsequent accumulation of stepwise resistance mutations, occurring over decades and prior to the explosion of HIV in this region, yielded MDR and XDR, permitting the emergence of compensatory mutations. Our results suggest that drug-resistant strains circulating today reflect not only vulnerabilities of current TB control efforts but also those that date back 50 y. In drug-resistant TB, isoniazid resistance was overwhelmingly the initial resistance mutation to be acquired, which would not be detected by current rapid molecular diagnostics employed in South Africa that assess only rifampicin resistance.

## Editors' Summary

**Background:**

Tuberculosis (TB)—a contagious bacterial disease that usually infects the lungs—is a global public health problem. Every year, about 9 million people develop active TB disease, and 1.5 million people die from the disease. *Mycobacterium tuberculosis*, the organism that causes TB, is spread in airborne droplets when people with TB cough. The symptoms of TB include cough, weight loss, and fever. Diagnostic tests for the disease include sputum smear microscopy (microscopic analysis of mucus coughed up from the lungs) and chest X-rays. TB can be cured by taking a regimen of multiple antibiotics daily for 6 mo. However, the emergence of multidrug-resistant tuberculosis (MDR-TB, TB with resistance to both isoniazid and rifampicin) and extensively drug-resistant tuberculosis (XDR-TB, MDR-TB with additional resistance to both quinolones and second-line injectable agents), together with the spread of HIV (which increases susceptibility to TB), is now threatening TB control efforts. MDR-TB is caused by *M*. *tuberculosis* strains that have acquired mutations (genetic changes) that make them resistant to isoniazid, rifampicin, and sometimes other anti-TB drugs; XDR-TB is caused by bacteria that are resistant to isoniazid, rifampicin, one or more fluoroquinolones (for example, ofloxacin), and at least one injectable second-line drug (for example, kanamycin).

**Why Was This Study Done?:**

A better understanding of the origins of drug-resistant TB is essential for effective control of TB. Public health experts need to know whether the emergence of drug-resistant TB is caused by inadequacies in TB control or related to other factors such as the spread of HIV and whether new resistant strains of *M*. *tuberculosis* repeatedly emerge during XDR-TB outbreaks or whether the transmission of a single drug-resistant strain drives these outbreaks. Here, the researchers use whole genome sequencing and dating analysis to investigate the origin and evolution of an XDR-TB outbreak identified in 2005 in Tugela Ferry, KwaZulu-Natal, South Africa. The predominant strain of XDR *M*. *tuberculosis* isolated during this large XDR-TB outbreak belongs to a subfamily called LAM4. Since the outbreak began, XDR-TB has also been reported in hospitals across KwaZulu-Natal, and some of these outbreaks have been caused by bacterial strains not falling within the LAM4 spoligotype (“spoligotyping” characterizes *M*. *tuberculosis* strains based on the presence of unique DNA sequences in a specific region of the bacterial genome).

**What Did the Researchers Do and Find?:**

The researchers tested the antibiotic susceptibility of 337 clinical isolates of *M*. *tuberculosis* collected in KwaZulu-Natal between 2008 and 2013 and of three historical isolates—two collected in the province in the mid-1990s and a third from the Tugela Ferry XDR outbreak. They sequenced the whole genome of these isolates and used comparative techniques to assess the isolates’ relatedness and to investigate the acquisition of drug resistance. This analysis revealed a 50-member clone of XDR bacteria among the isolates collected across KwaZulu-Natal that was highly related to the LAM4 strain (a clone is defined here as a set of strains in which each member differs by no more than ten single nucleotide polymorphisms [SNPs] from at least one other member; an SNP is a type of genetic variant). Mutations that conferred isoniazid resistance in this clone were acquired in about 1957; MDR and XDR strains emerged in about 1984 and 1995, respectively. The analysis also indicates that MDR and XDR evolved de novo 56 times and nine times, respectively, and that isoniazid resistance nearly always evolved before rifampicin resistance.

**What Do These Findings Mean?:**

These findings provide new information about the ordering and timing of the acquisition of drug-resistance mutations by *M*. *tuberculosis* in KwaZulu-Natal but do not necessarily represent the evolution of XDR-TB in other settings. Most notably, these findings indicate that the ancestral precursor of the Tugela Ferry XDR outbreak strain gained resistance to first-line antibiotics shortly after these antibiotics became available for clinical use. Subsequent stepwise accumulation of additional resistance mutations that occurred over decades led to the emergence of MDR and XDR strains. Importantly, the emergence of these strains occurred before the explosion of HIV in KwaZulu-Natal. Thus, these findings highlight the dire repercussions of the failure of historic attempts to control resistance to first-line anti-TB drugs and draw attention to the need for new anti-TB drugs to be used prudently to prevent early fixation of resistance and to protect the useful lifespan of these agents. Finally, the finding that isoniazid resistance is a key initiation event for progression to MDR and XDR suggests that TB control programs should test routinely for both isoniazid and rifampicin resistance to ensure early detection of drug-resistant TB.

**Additional Information:**

This list of resources contains links that can be accessed when viewing the PDF on a device or via the online version of the article at http://dx.doi.org/10.1371/journal.pmed.1001880.

- The World Health Organization (WHO) provides information (in several languages) on TB and on MDR-TB; the Global Tuberculosis Report 2014 provides information about TB around the world; a supplement to the report entitled “Drug-Resistant TB—Surveillance and Response” is available
- The Stop TB Partnership is working towards TB elimination and provides personal stories about TB (in English and Spanish)
- The United States Centers for Disease Control and Prevention provides information about TB and about drug-resistant TB (in English and Spanish)
- The US National Institute of Allergy and Infectious Diseases also has detailed information on TB, including a drug-resistant TB visual tour
- TB & Me, a collaborative blogging project run by patients being treated for MDR-TB and Mèdecins sans Frontiéres, provides more patient stories
- The not-for-profit organization Global Health Education provides information about TB in South Africa
- MedlinePlus has links to further information about TB (in English and Spanish)

## Introduction

The global burden of tuberculosis (TB) remains high, with an estimated 9 million active disease cases and 1.5 million deaths in 2013 [1]. Multidrug-resistant (MDR) TB, defined as *Mycobacterium tuberculosis* with in vitro resistance to both isoniazid and rifampicin, accounted for at least 480,000 incident cases and 210,000 attributed deaths in 2013 [1]. Extensively drug-resistant (XDR) TB, which is MDR with additional resistance to both quinolones and second-line injectable agents [2], has been reported in 100 countries [1]. With high morbidity, XDR poses a dire threat to public health, particularly in populations with high HIV prevalence [1,3].

The incidence of TB in South Africa is estimated by the WHO to be 860 (776–980) per 100,000 population, which is among the highest in the world [1]. With a population of approximately 10 million, KwaZulu-Natal is the easternmost of South Africa’s nine provinces. While its provincial TB incidence is similar to that of the rest of South Africa (889 per 100,000 in 2012, based on treatment initiation data) [4], KwaZulu-Natal has been notable for disproportionately high rates of drug-resistant TB [4,5]. Compounding this epidemic, South Africa has seen a dramatic increase in HIV prevalence in the last 25 y. The Joint United Nations Programme on HIV and AIDS (UNAIDS) estimates that national adult HIV prevalence was only 0.3% in 1990 but rose to 19.1% in 2013 [6]. In KwaZulu-Natal, HIV rates are particularly high, with 37.4% HIV prevalence documented among pregnant women in 2011 [4].

In 2005, the identification of an outbreak of XDR-TB at the Church of Scotland Hospital in KwaZulu-Natal, Tugela Ferry, raised global alarm and called attention to the prospect of dissemination of potentially untreatable TB [7]. Not only was resistance to four or more classes of antibiotics noted in these strains, but also the disease, in the context of HIV coinfection, was rapidly fatal, with 98% mortality [7]. Traditional genotyping by IS6110 fingerprinting and spoligotyping identified a predominant strain of global *M*. *tuberculosis* lineage 4 and spoligotype, ST60, later termed LAM4/F15/KZN (henceforth referred to as LAM4), suggestive of a clonal outbreak of a single drug-resistant strain [7–11]. Targeted sequencing of resistance mutations in a subset of these XDR strains revealed identical mutations [9], further supporting the theory of acquisition of XDR-level resistance and subsequent transmission. Nosocomial spread was deemed likely by a social network analysis [9].

Since the events at Church of Scotland Hospital, which still stands as the largest outbreak of XDR-TB ever reported, XDR-TB has been reported in the majority of hospitals across KwaZulu-Natal [12], and more than 516 XDR-TB cases have been reported within Tugela Ferry alone [13]. In addition, XDR-TB caused by strains not falling within the LAM4 spoligotype have been seen, indicating repeated evolutionary emergences of XDR among strains circulating within the region [10,14]; however, the relative contribution of de novo versus vertically inherited resistance of XDR-TB is unknown. It is also unknown how and when XDR-level drug resistance developed, information that could be exploited to detect and prevent higher-level resistances from emerging in South Africa and elsewhere in the world.

While accumulation of drug-resistance mutations can confer a fitness cost to bacteria, subsequent development of compensatory mutations can ameliorate these costs by restoring certain affected physiological functions while maintaining drug resistance [15–17]. Identification of compensatory mutations among clinical strains of *M*. *tuberculosis* [18–20] has improved our understanding of drug resistance and fitness, but this area remains incompletely studied.

Whole genome sequencing efforts that target large collections of *M*. *tuberculosis* have provided critical insights into *M*. *tuberculosis* population dynamics, including *M*. *tuberculosis* transmission and the molecular causes of drug resistance [20–23]. Although some strains from KwaZulu-Natal have been sequenced [24,25], there has been no large-scale sequencing project from this province or studies that have systematically addressed the molecular evolution of XDR. In the largest compilation of whole genome sequences from clinical isolates of *M*. *tuberculosis* from South Africa, we used a combination of comparative genomic techniques to elucidate when and how epidemic XDR drug resistance emerged. With knowledge of a strain’s date of collection, determination of the number of single nucleotide polymorphism (SNP) differences between sequenced strains, and the estimated mutation rate of *M*. *tuberculosis*, we were able to utilize Bayesian [26] inference to estimate the dates of acquisition of resistance mutations within the Tugela Ferry ancestor. We discuss the implications of these findings with respect to current and future TB control.

## Methods

### Specimen Collection and Characterization

We selected 337 clinical isolates of *M*. *tuberculosis* with diverse drug susceptibility patterns. Strains were collected both retrospectively and prospectively from 2008 to 2013 from all 11 districts of KwaZulu-Natal (Table 1). Strains were chosen for study inclusion on the basis of a predetermined drug resistance pattern so that the dataset was heavily weighted toward drug-resistant strains rather than accurately reflecting the epidemiology of the region. Written informed consent was obtained from study participants prior to cohort enrollment. Biomedical Research Ethics Council (BREC) approval from the University of KwaZulu-Natal was granted for whole genome sequencing of clinical strains. On all study isolates, drug susceptibility testing (DST) was performed by the critical concentration method, using the WHO recommended concentrations [27]. The following drugs were assayed in all strains, with their respective critical concentration in parentheses (in μg/mL): rifampicin (1.0), isoniazid (0.2 and/or 1.0), streptomycin (2.0), kanamycin (6.0), and ofloxacin (2.0). Extended DST was performed for key isolates (Table 1) with the following drugs: capreomycin (10.0), ethambutol (7.5), and ethionamide (10.0). Pyrazinamide resistance testing was performed using PZA MGIT (100.0) or Nicotinamide (500.0). Subject data included age, gender, AFB smear, and HIV status, when available. Study participants were assigned GPS coordinates corresponding to their home provincial district or site of sputum collection.

**Table 1:** Description of study isolates. The 337 clinical study isolates derived from five patient cohorts and were both prospectively and retrospectively collected from all 11 districts of KwaZulu-Natal from 2008 to 2013. Culture conditions describe the initial *M*. *tuberculosis* isolation method from sputum. If subsequent single colony isolation (SCI) was performed prior to DNA extraction on the entire study cohort or a subset of the cohort, then this is denoted. Drugs for which DST was performed are abbreviated as follows: rifampicin (R), isoniazid (H), nicotinamide (N), pyrazinamide (P), ethambutol (E), streptomycin (S), kanamycin (K), ofloxacin (O), ethionamide (Et), and capreomycin (C).

Cohort Name	Cohort Description	Collection Strategy and Year(s) of Collection	Number of Study Isolates	Culture Conditions	Drug Susceptibility Testing
KwaZulu-Natal Drug Surveillance Study (KZNSUR) [28]	Cross-sectional study of outpatients and hospitalized inpatients with cough. Samples were collected from each district in KwaZulu-Natal.	Retrospective, 2008–2010	90	7H10, 7H11; SCI entire cohort	R, H, E, N, S, K, O, Et, C
Prospective Collection of Extensively Drug-Resistant TB (PROX) [29]	Prospective collection of patients newly initiating XDR therapy at the central TB hospital in KwaZulu-Natal (King DinuZulu Hospital).	Prospective, 2010–2012	53	7H10, 7H11; SCI entire cohort	R, H, E, N, S, K, O, Et. P, C
Phage Study [30]	Patients newly diagnosed with pulmonary TB at the major outpatient clinic in central Durban (Prince Cyril Zulu) prior to initiation of treatment.	Prospective, 2013	61	MGIT; SCI subset	R, H, E, S, K, O
National Health Services Laboratory (NHLS)	Collection of drug-resistant clinical isolates sent for DST at the central National Health Laboratory Services TB Laboratory.	Prospective, 2013	103	MGIT; 7H10; SCI entire cohort	R, H, S, K, O
Collection of Urine Blood Sputum Study (CUBS)	Prospective collection of patients newly initiating MDR or XDR therapy at the central TB hospital in KwaZulu-Natal (King DinuZulu Hospital)	Prospective, 2013	30	7H10; SCI subset	R, H, E, S, K, O

We also selected for sequencing three historical strains previously collected in KwaZulu-Natal for resequencing [25,24]: KZN4207 (drug susceptible, collected in Durban in 1995), KZN1435 (MDR, collected in Durban in 1994), and KZN605 (XDR, collected in Tugela Ferry in 2005).

### Whole Genome Sequencing

Genomic DNA was extracted using published methods [31]. The majority of strains were single colony selected prior to DNA isolation (S1 Methods and S1 Table). Library preparation and whole genome sequencing (WGS) were performed as previously described on the Illumina HiSeq 2000 at the Broad Institute [32]. The median depth of sequencing was 143x, and coverage of the H37Rv genome was 99.9%. Sequencing data were submitted to the Sequence Read Archive NCBI under the following umbrella BioProject identifiers: PRJNA183624 and PRJNA235615.

### Bioinformatic Analysis

#### Primary analysis

Reads were mapped onto a reference strain of H37Rv (GenBank accession number CP003248.2) using BWA version 0.5.9 [33]. In cases in which read coverage of the reference was greater than 200x, reads were down-sampled using Picard [34] prior to mapping. Positions that varied relative to the reference were identified using Pilon version 1.5 as described [32].

#### Strain diversity and biogeography

We conducted phylogenetic analyses for both the entire set of 340 strains, as well as for a subset of 111 strains belonging to the LAM4 spoligotype. For each set, all sites with unambiguous SNPs in at least one strain were combined into a concatenated alignment. Ambiguous positions were treated as missing data. The concatenated alignment was then used to generate a midpoint rooted phylogenetic tree in RAxML (version 7.3.3) [35] under a GTRCAT substitution model with 1,000 bootstrap replicates. Global *M*. *tuberculosis* lineage designations were assigned based on phylogeny and regions of difference [36]. Each strain’s “digital” spoligotype was predicted by statistically testing for the presence of each of 43 unique spacer sequences used in classical spoligotyping from sequence reads. Results were matched to spacer pattern profiles at SITVITWEB to generate a named spoligotype (S1 Methods) [37]. Clonal strains were identified using a density-based clustering algorithm [38] that grouped strains that differ by no more than ten SNPs to at least one other member within a clone (S1 Methods) [39–41].

Mantel tests were performed to evaluate the relationship between genetic and geographic distances among strains using the ZT software v1.1 [42]. Pairwise genetic distances were calculated as the number of SNP differences between strains, and geographic distances were calculated using the haversine formula [43] and points of origin for strain pairs.

#### Ordering and dating evolution of drug resistance

A curated list of genomic polymorphisms associated with drug resistance was defined for each tested drug based on a literature review (S1 Methods). Polymorphisms associated with compensatory mechanisms to isoniazid, rifampicin, and ethambutol were also defined (S1 Methods). Strains with predicted resistance were identified based on the carriage of mutations from the curated list. We used PAUP [44] to reconstruct the patterns of drug resistance mutation gains and losses throughout the phylogenetic tree representing all 340 strains. PAUP was run using a cost matrix that assigned a 10x greater cost for a loss event relative to a gain event. We used BEAST [26] to estimate a mutation rate and to determine dates for the acquisition of mutations within the LAM4 spoligotype.

BEAST was run for 50 million iterations, sampling every 1,000 iterations, using the relaxed lognormal clock (uncorrelated) model. The relaxed molecular clock model assumes independent rates on different branches, which was consistent with previously published reports [45], as well as initial BEAST analyses that we conducted involving lineages 2 and 4, indicating that there may be substantial variation in evolutionary rates within *M*. *tuberculosis*. In addition, since the BEAST statistic “ucld.stdev” was greater than zero (0.189) for our dataset, this indicated that our data did exhibit rate heterogeneity within the LAM4 spoligotype. The first 5 million iterations were excluded as “burn-in.” We used the GTR + Gamma substitution model, estimated base frequencies, and the “Gamma + invariant sites” site heterogeneity model. We enforced the topology of the SNP-based tree determined using RAxML [35]. We used a starting value for the mean mutation rate of 0.35 SNPs/genome/year [39,41,46–48]. We assayed a range of values for the starting mean mutation rate, covering the range of values previously reported in the literature, with little difference in the output. BEAti was used to construct the BEAST input file, and default values were used for all other priors. The program Tracer was used to examine mixing and effective sample size (ESS) in order to assess chain length and model convergence. ESS indicates the number of effectively independent draws from the posterior distribution to which the Markov chain is equivalent. A low ESS for a particular parameter (ESS < 100) would indicate that the trace contained a lot of correlated samples and thus may not well represent the posterior distribution. In our analysis, all statistics had an ESS greater than 150. The results were consistent across several runs of the same model. Estimated dates are given with 95% highest posterior density (HPD) intervals.

## Results

Our study included 337 participants with an average age of 33.8 y and a standard deviation of 10.7 y, of whom 165 (49%) were male (Table 2). Overall, 140 patients were HIV positive, 51 were HIV negative, and 146 had unknown HIV status. Baseline characteristics were similar among HIV-positive and HIV-negative individuals, with the exception that HIV-negative individuals were younger (*p* = 0.0030), more likely to be smear positive (*p* = 0.0139), and more likely to live outside of eThekwini, the provincial capital (*p* = 0.0132).

**Table 2:** Demographic characteristics of participants and phenotypic drug susceptibility of strains. Data are *n* (%) or mean ± standard deviation (SD).

	HIV Positive	HIV Negative		HIV Unknown	All Patients
Characteristic	(*N* = 140)	(*N* = 51)	*p*-value*	(*N* = 146)	(*N* = 337)
**Age (y); mean ± SD**	34.0 ± 8.5	31.8 ± 13.4	0.0030	34.3 ± 11.5	33.8 ± 10.7
**Male**	65 (46)	30 (59)	0.1435	70 (48)	165 (49)
**Smear status**					
** smear positive**	89 (64)	42 (82)	0.0139	51 (35)	182 (54)
** smear negative**	50 (36)	9 (18)	0.0209	24 (16)	83 (25)
** smear unknown**	1 (1)	0 (0)	1.0000	71 (49)	72 (21)
**Phenotypic DST results**					
** Susceptible**	39 (28)	22 (43)	0.0542	27 (18)	88 (26)
** Monodrug resistant**	7 (5)	2 (4)	1.0000	14 (10)	23 (7)
** Polydrug resistant**	4 (3)	0 (0)	0.5751	15 (10)	19 (6)
** MDR sensu stricto**	54 (39)	16 (31)	0.3996	70 (48)	140 (42)
** XDR**	36 (26)	11 (22)	0.7044	20 (14)	67 (20)
** Tugela Ferry XDR** ^†^	27 (75)	8 (73)	0.6750	14 (70)	49 (73)
** other XDR** ^†^	9 (25)	3 (27)	1.0000	6 (30)	18 (27)
**Geography**					
** eThekwini**	73 (52)	36 (71)	0.0312	69 (47)	178 (53)

* HIV-positive and HIV-negative individuals were compared using Fisher’s exact test for categorical variables and nonparametric Mann-Whitney test for continuous variables.

^†^ Data in this row are from the XDR data, not the total dataset.

Results having a *p*-value < 0.05 were considered statistically significant.

A clinical sample was obtained from each patient; *M*. *tuberculosis* was isolated using standard approaches and phenotypic DST was performed on each isolate using standard methodology (S1 Methods). Phenotypic DST revealed 88 susceptible, 23 monodrug-resistant (defined as phenotypic resistance to only one drug), 19 polydrug-resistant (defined as phenotypic resistance to two drugs that does not meet criteria for MDR), 140 MDR sensu stricto, and 67 XDR *M*. *tuberculosis* strains (Table 2). Phenotypic MDR and XDR-TB cases were identified in all 11 districts of KwaZulu-Natal. While we observed a trend toward HIV-negative individuals harboring more drug-susceptible TB, this observation did not meet statistical significance (*p* = 0.0542).

**Fig 1:**
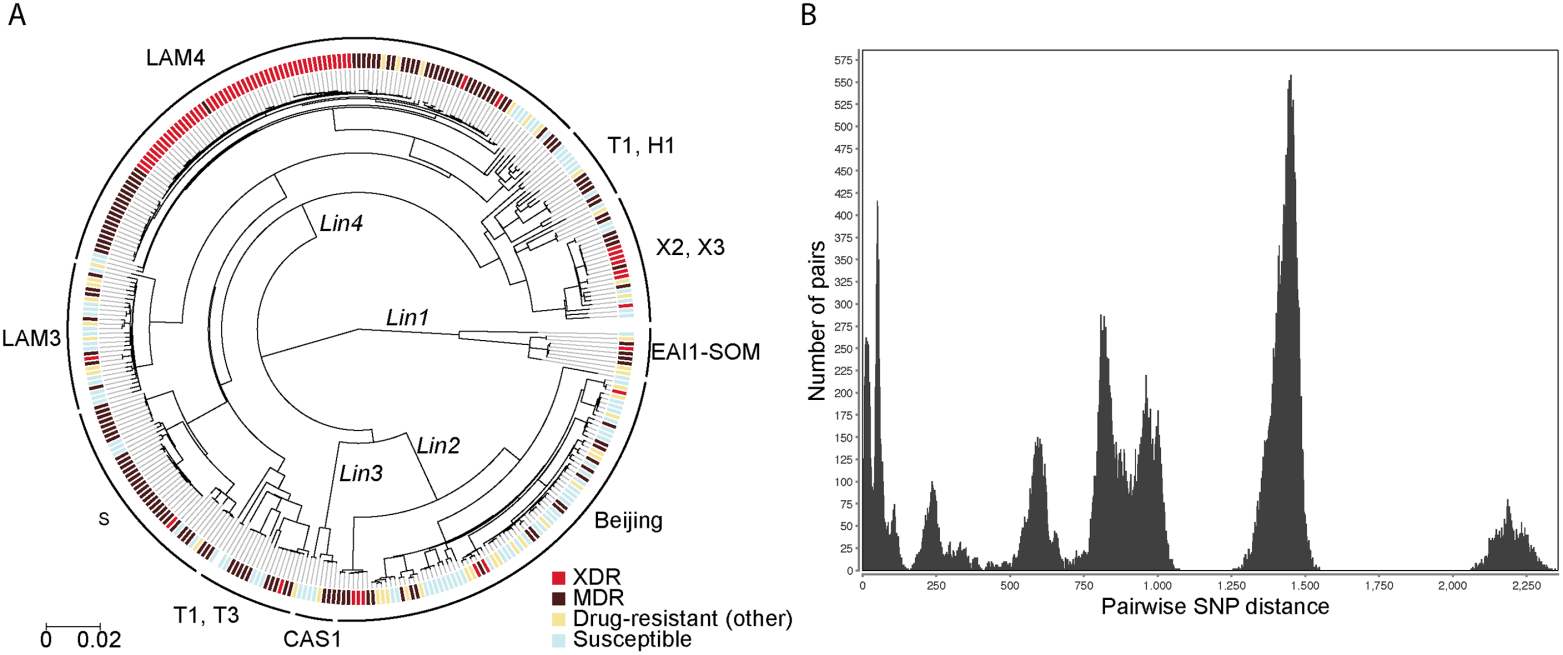
Diverse strains contribute to drug resistance in KwaZulu-Natal. (A) Midpoint rooted maximum-likelihood phylogeny of 340 *M*. *tuberculosis* isolates. Four of the seven known *M*. *tuberculosis* lineages were identified: CAS (*Lin1*), Beijing (*Lin2*), EAI (*Lin 3*), and Euro-American (*Lin4*). Digital spoligotyping identified 17 unique spoligotypes in the dataset; spoligotypes are shown on this figure if they are represented by three or more strains. Corresponding spoligotypes and phenotypes are reported for all strains in S4 Table. Phenotypic XDR, MDR, poly- and monodrug resistance (labeled “Drug-resistant other”), and pansusceptible strains are indicated by colored tick marks at the tip of each leaf node. (B) Histogram of pairwise SNP distances between strains. The number of pairs within each SNP distance range is plotted. The peaks correspond to the distance between major lineages. The peak at the far left of the figure corresponds to the distance between pairs of strains within a clone.

We performed WGS on all 337 clinical strains, as well as on three historical strains isolated prior to the study collection period. We assessed the diversity and phylogenetic relatedness among strains using information from 17,232 variable sites with SNPs relative to the H37Rv reference genome (Fig 1; S1 Fig). The resulting phylogenetic tree revealed four of the seven main global lineages of *M*. *tuberculosis* [36,49–51] to be circulating in KwaZulu-Natal during the sampling time frame. The vast majority of isolates (95%) belonged to lineages 2 and 4, with lesser representation from lineages 1 and 3.

**Fig 2:**
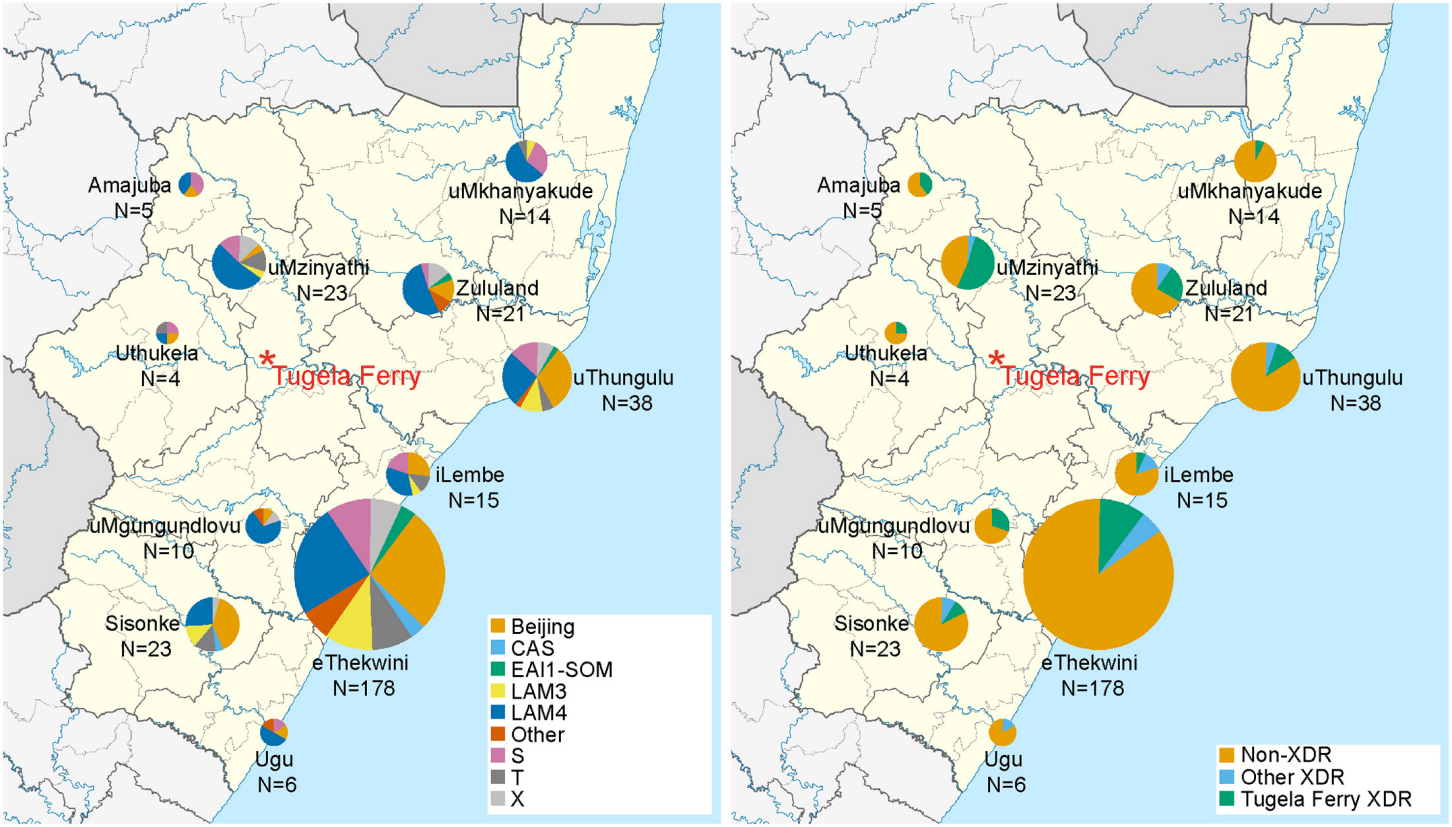
Wide geographic spread of diverse strains across KwaZulu-Natal and wide distribution of XDR and the Tugela Ferry XDR Clone members. A map of the 11 districts of KwaZulu-Natal [52] is shown in which pie charts indicate (A) the fraction of sequenced *M*. *tuberculosis* belonging to computationally predicted spoligotypes (see key). In (B), the fraction of strains with a phenotypic classification of XDR and membership in the Tugela Ferry XDR Clone are represented (see key). The size of the pie chart indicates the relative number of strains sequenced from each of the 11 districts within KwaZulu Natal. Tugela Ferry, in the uMzinyathi district, is indicated in red.

A computational or digital spoligotype prediction was performed, and 17 unique spoligotypes were identified (S1 Table) [37]. Spoligotype diversity was well represented in all districts of KwaZulu-Natal (Fig 2, panel A). Using a Mantel test, we determined that there was very low correlation between geographic and genetic distances among strains (r = -0.067906, *p* = 0.001760), indicating that strains did not cluster geographically. Older transmission events and/or high patient mobility between districts likely account for this pattern.

We defined a “clone” as a set of strains in which each member differs by no more than ten SNPs to at least one other member, which is similar to definitions used in previous genomic studies of *M*. *tuberculosis* transmission (S2 Fig, S1 Methods) [39–41]. Nearly one-third of the strains (107 of 340, 31%) belonged to 11 such clones (S2 Table), which were distributed across six spoligotypes and three lineages (S3 Fig). All clones were phenotypically drug resistant, indicating recent person-to-person spread of a diverse set of drug-resistant strains that included both HIV-positive and HIV-negative individuals.

**Fig 3:**
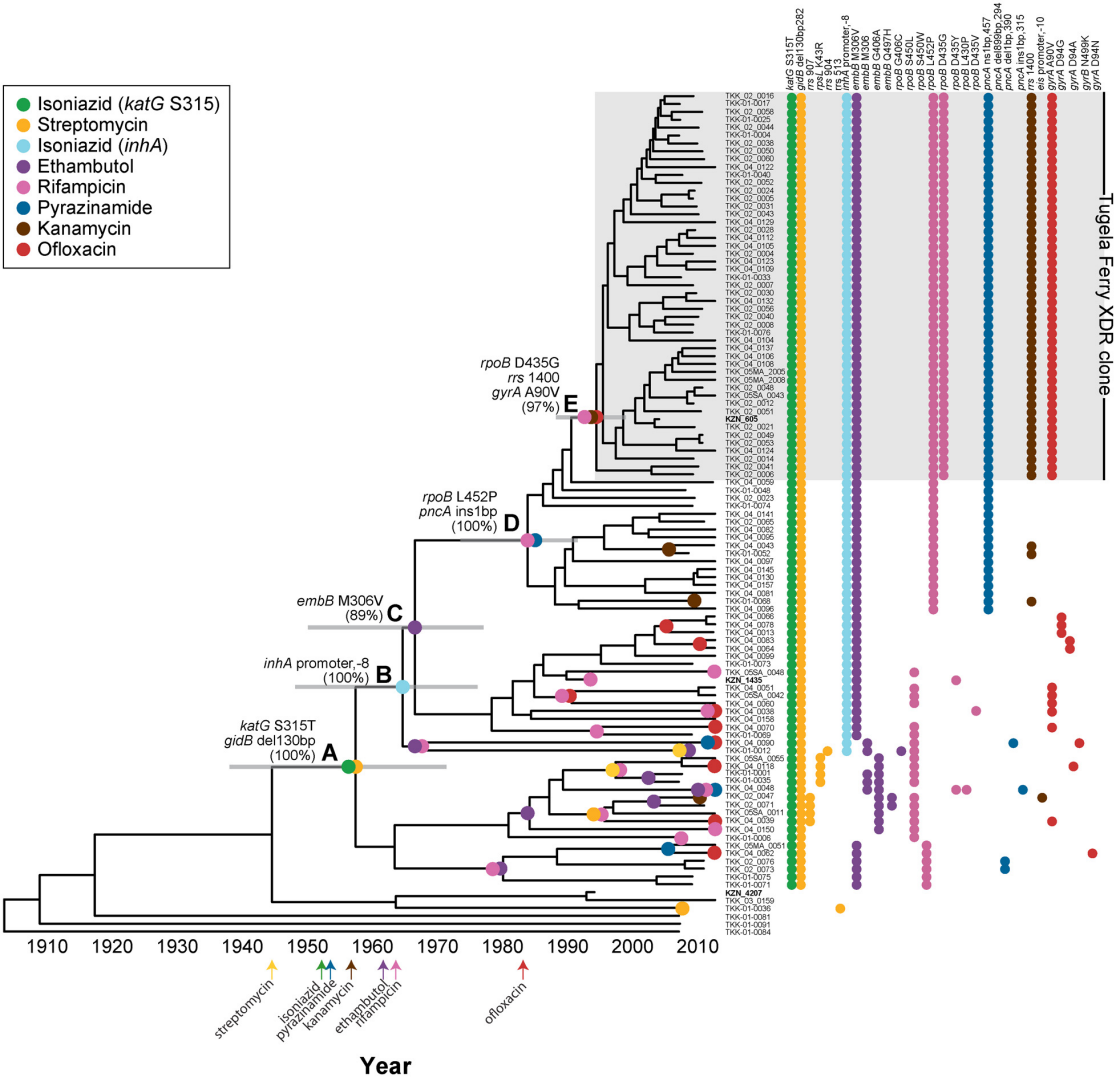
Molecular evolution and dating of drug resistance emergence within the Tugela Ferry XDR Clone. Midpoint rooted maximum-likelihood phylogeny of 107 *M*. *tuberculosis* isolates of the LAM4 spoligotype. The gray shaded box identifies the Tugela Ferry XDR Clone. KZN605, the historical XDR strain collected in Tugela Ferry during the outbreak, is a member of this clone. Two additional historical isolates, KZN1435 and KZN4207, are not members of the Tugela Ferry XDR Clone. Each evolutionary gain of a drug resistance mutation was assigned to its position on the phylogenetic tree by parsimony (colored circles). A–E traces the stepwise order of drug resistance acquisition in the Tugela Ferry XDR Clone and estimates the year when each mutation was gained. Gray bars indicate the 95% highest posterior density (HPD) intervals. (A) *katG* S315T (isoniazid); *gidB* 130 bp deletion (streptomycin); 1957 (95% HPD: 1937–1971); (B) *inhA* promoter -8 (isoniazid and ethionamide); 1964 (95% HPD: 1948–1976); (C) *embB* M306V (ethambutol); 1967 (95% HPD: 1950–1978); (D) *rpoB* L452P (rifampicin); *pncA* 1bp insertion (pyrazinamide); 1984 (95% HPD: 1974–1992); and (E) *rpoB* D435G (rifampicin); *rrs* 1400 (kanamycin); *gyrA* A90V (ofloxacin); 1995 (95% HPD: 1988–1999). The accumulation of individual drug-resistant mutations within a strain is denoted to the right of the phylogenetic tree. The dates of drug discovery are displayed at the bottom of the figure [53]. Four additional LAM4 strains on a distant branch were not included in this figure because of size constraints. Bootstrap values are provided for lettered nodes, and bootstrap values for all nodes are shown in S5 Fig.

The “historical” Tugela Ferry XDR strain, KZN605, was nested phylogenetically within a large clone of 50 LAM4 strains with predominantly phenotypic XDR (Fig 3). All of the strains within this clone (henceforth referred to as the Tugela Ferry XDR Clone) possessed the characteristic drug resistance mutations that were previously identified in XDR-TB strains circulating in Tugela Ferry during the outbreak [9,24], further indicating this clone’s continued prevalence within KwaZulu-Natal. Patients in whom the Tugela Ferry XDR Clone was isolated were from ten of the 11 districts within the province (Fig 2, panel B). In addition, the Tugela Ferry XDR Clone was not overrepresented among HIV-positive patients (*p* = 0.6750) (Table 2). This suggests that strains within this clone were neither geographically constrained nor restricted to immunodeficient hosts.

Many of the sequenced LAM4 strains were closely related to the Tugela Ferry XDR Clone but had different DST profiles (Fig 3 and S2 Fig), giving us an opportunity to finely dissect the order of acquisition of mutations giving rise to the Tugela Ferry XDR Clone [9,24]. LAM4 strain phylogeny was recalculated using data from only LAM4 strains, and parsimony was used to place the origin of known resistance-conferring mutations on the tree. The recalculated LAM4 tree was consistent with our previous tree containing data from all strains with all key internal nodes involved in the evolution of drug resistance having bootstrap values greater than 89%. This enabled us to confidently assign evolutionary ordering of drug resistance mutation acquisition (Fig 3 and S5 Fig).

As shown in Fig 3, the first step towards XDR-level resistance in this epidemic clone was the acquisition of isoniazid and streptomycin resistance-conferring mutations in *katG* and *gidB*, respectively, which were gained at node A of the phylogenetic tree (100% bootstrap support). With accumulation of successive mutations, the ancestral strain (and its descendants) gained (i) additional polydrug resistance to ethionamide and ethambutol via mutations in the *inhA* promoter and *embB* (nodes B and C, respectively, 100% and 89% bootstrap support), (ii) MDR via mutations in *rpoB* and *pncA* that conferred resistance to rifampicin and pyrazinamide (node D, 100% bootstrap support), and (iii) XDR via mutations in *rrs* and *gyrA*, which conferred resistance to kanamycin and ofloxacin, respectively, and an additional *rpoB* mutation (node E, 97% bootstrap support). This ordering was highly supported by bootstrapping (all key nodes had bootstrap values ≥89%) in the phylogenetic reconstruction. Thus, the first step towards XDR-level drug resistance in this epidemic clone was the acquisition of isoniazid and streptomycin resistance followed by ethambutol and ethionamide resistance, then rifampicin and pyrazinamide resistance, and, ultimately, kanamycin and ofloxacin resistance.

Because we had dates of isolation for all sequenced strains—including strains that were isolated more than 20 y ago—we applied a Bayesian statistical approach to estimate when mutations leading to the Tugela Ferry XDR Clone emerged. Using this approach, which takes into account the phylogeny of LAM4 strains, the dates of their isolation, and published mutation rates for *M*. *tuberculosis* [39,41,46–48], we calculated that LAM4 in KZN mutated at a rate of 0.61 SNPs/genome/year. This mutation rate was higher than other previously published mutation rates, regardless of which rate from the literature was used as the starting mean value. Applying this rate, we estimated that drug resistance mutations at node A were acquired in 1957 (95% HPD: 1937–1971), soon after streptomycin and isoniazid were developed. MDR-level resistance was acquired in 1984 (95% HPD: 1974–1992; node D), and XDR-level resistance was acquired in 1995 (95% HPD: 1988–1999; node E), 10 y prior to its acute recognition in 2005 in Tugela Ferry (Fig 3). The dating analysis within the LAM4 spoligotype consistently assigned drug resistance gains after the drug discovery date, indicating that drug resistance emergence in the region mirrored the dates of drug discovery.

We also observed multiple drug resistance mutations within LAM4 that emerged outside the Tugela Ferry XDR Clone (Fig 3). Many of these mutations were acquired at leaf nodes, which implied very recent gains of resistance. Including the Tugela Ferry ancestor, we calculated that genotypic MDR sensu stricto—defined as both isoniazid and rifampicin resistance-conferring mutations—independently arose a minimum of 13 times. Within LAM4, the Tugela Ferry XDR Clone represented the single and only evolutionary gain of genotypic XDR—as defined by acquisition of resistance-conferring mutations to the four XDR-defining drugs: isoniazid, rifampicin, ofloxacin, and kanamycin. However, within LAM4 we also observed ten independent gains of either a kanamycin or an ofloxacin resistance-conferring mutation in a background of genotypic MDR sensu stricto. As such, 13 LAM4 strains identified in this study would be considered genotypic “pre-XDR” and only one SNP away from XDR-level resistance.

Beyond LAM4, we observed many other independent evolutionary emergences of MDR and XDR across this dataset. Twelve and seven spoligotypes contained strains with phenotypic MDR and XDR, respectively (S3 Table), suggesting that these resistance patterns emerged no fewer than 12 and 7 times. However, when we quantified the total number of independent evolutionary emergences of genotypic MDR and XDR across our entire dataset, we estimated that MDR sensu stricto and XDR evolved no less than 56 and nine independent times, respectively (S3 Table).

**Fig 4:**
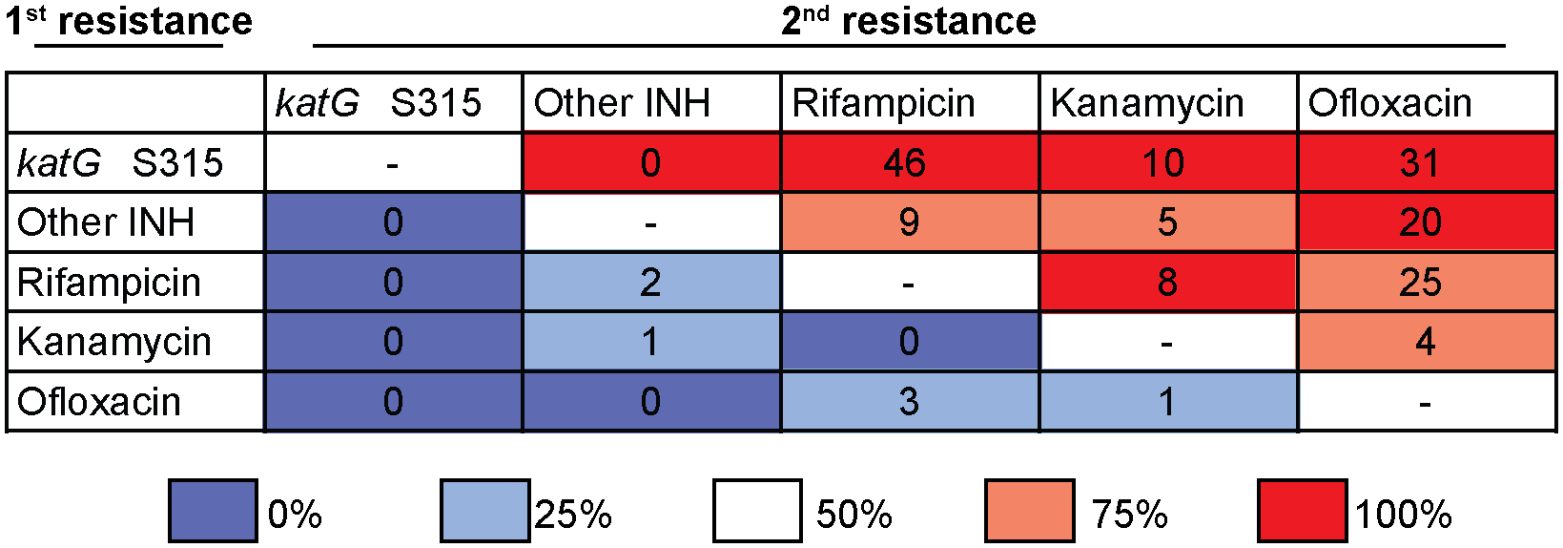
Isoniazid resistance is the first step towards drug resistance. Acquisition of *katG* S315 mutations precedes all other resistance mutations, including rifampicin, in all instances in which the order of acquisition can be disambiguated. For the 214 strains with genotypic resistance to two or more MDR or XDR defining drugs, and in which the order of acquisition of these mutations could be disambiguated, we quantified the number of evolutions in which resistance to one drug was gained before resistance to a second drug. Isoniazid resistance was divided into mutations conferred by the *katG*S315 codon versus “Other INH” mutations (defined as loss-of-function mutations in *katG* that do not involve codon 315 or mutations in the *inhA* promoter). Reported numbers represent the number of independent evolutionary events (not the number of strains) in which the drug resistance indicated by the row labeled “first resistance” was acquired before the drug resistance indicated by the column labeled “second resistance.” The background color is shaded to indicate the fraction of unambiguous evolutionary events in which the “first resistance” was acquired before the “second resistance” for that given drug pair.

Remarkably, the first drug resistance acquisition in the Tugela Ferry XDR Clone was consistent with other emergences of MDR and XDR across the entire dataset. For the 214 strains with genotypic resistance to two or more of the MDR and XDR defining drugs, we quantified the number of evolutions in which a specific drug resistance mutation was gained before a second resistance mutation. We observed that isoniazid resistance via nonsynonymous mutation at the *katG* S315 codon was gained before rifampicin resistance in 46 unique evolutionary events, whereas rifampicin resistance was never acquired before the *katG* S315 mutation (Fig 4). When we repeated this for all pairwise comparisons, we found that isoniazid resistance, conferred by mutation of the *katG* S315 codon, preceded or co-occurred with resistance mutations to all other drugs in our dataset. Mutations other than the *katG* S315 mutations that confer isoniazid resistance (i.e., *inhA* promoter mutations or *katG* deletions) occurred before rifampicin resistance mutations in nine unique events, whereas we only observed the reverse ordering twice. These data indicate that, beyond the Tugela Ferry XDR Clone, isoniazid resistance, and in particular the S315 codon mutation in *katG*, has been the initial resistance-conferring mutation leading to polydrug resistance, including MDR and XDR, among strains from KwaZulu-Natal.

As in other organisms, in vitro studies have suggested that drug resistance in *M*. *tuberculosis* may be associated with a variable fitness cost that can be offset by compensatory evolution [18,15]. Nonsynonymous mutations in the α and β’ subunits of RNA polymerase have been postulated to compensate for fitness costs associated with rifampicin resistance [15,19]. Among our 226 strains with phenotypic rifampicin resistance, 76 strains had mutations known to compensate for rifampicin resistance (S4 Table) [20,19,54,55]. Using the phylogenetic framework and parsimony, we determined that 23 of the 27 previously described compensatory mutations had an evolutionary pattern consistent with rifampicin compensation, i.e., mutations that evolved only after or concurrent with mutations that conferred rifampicin resistance.

We also attempted to identify novel rifampicin compensatory mutations with this approach. In addition to the 27 previously described mutations, we detected an additional 38 nonsynonymous polymorphisms in *rpoA*, *rpoC*, and the non-rifampicin resistance-determining regions (RRDR) regions of *rpoB* (S4 Table). By parsimony analysis, we established the acquisition order of these *rpoA*, *rpoC*, and non-RRDR *rpoB* mutations in relation to genotypic rifampicin resistance. An additional 26 of these previously uncharacterized mutations also evolved in a pattern consistent with a role in compensation, which suggests that they may also function in this capacity. While there were ten unique RRDR mutations with subsequent or concurrent evolutionary gain of a putative rifampicin compensatory polymorphism, the vast majority of putative compensatory mutations occurred in association with *rpoB* S450L (*p <* 0.001) (S5 Table). This pattern was observed regardless of whether the compensatory mutation was previously known or uncharacterized.

Beyond rifampicin compensation, we also applied our combined phylogenetic and parsimony approach to known isoniazid and ethambutol compensatory mutations. With respect to isoniazid compensation, only a single evolution of the *ahpC* promoter mutation was observed in our dataset (S6 Table). It was gained after genotypic isoniazid resistance, which supports its compensatory role for certain isoniazid resistance mechanisms [41]. Nonsynonymous mutations in *ubiA* (Rv3086c) have previously been implicated in ethambutol resistance [56]. In our dataset, there were at least two occasions in which these mutations unambiguously arose prior to the acquisition of genotypic ethambutol resistance, suggesting that these are more likely to be stepping-stone mutations rather than compensatory (S6 Table).

## Discussion

We report on the WGS and comparative analysis of the largest collection of drug-resistant *M*. *tuberculosis* sequenced to date from South Africa. From analysis of these genomes, we determined the molecular antecedents of the Tugela Ferry XDR Clone and dated the emergence of genotypic resistance to eight drugs. We showed that the development of XDR in KwaZulu-Natal had its roots in first-line drug resistance that arose in the late 1950s and MDR that emerged in the 1980s. Our dating analysis indicated that the Tugela Ferry XDR Clone took nearly four decades to evolve from its initial isoniazid and streptomycin resistances to full-blown XDR. Although our data confirmed that the XDR outbreak in KwaZulu-Natal was indeed a clonal event, we showed that drug resistance in this region is driven by both the development of de novo drug resistance and clonal spread. We elucidated common evolutionary patterns of drug resistance acquisition and determined that isoniazid was overwhelmingly the first drug resistance to be acquired. Lastly, we validated that certain previously described rifampicin compensatory mutations do indeed evolve in a pattern consistent with compensation and have identified 26 novel polymorphisms that may also function in this capacity. Collectively, these data have important implications for the public health control of TB in sub-Saharan Africa and elsewhere.

Using a combination of likelihood, parsimony, and Bayesian computational approaches, we observed a decades-long evolutionary trajectory toward XDR-level drug resistance in LAM4 that mirrored the order and timing of introduction of antitubercular drugs into clinical practice [57]. Though parsimony-based approaches can interpret rapid independent evolution of an identical polymorphism in multiple strains as a single evolutionary event (occurring at a single node), our predictions indicated that resistance-conferring mutations evolved only after each drug’s clinical introduction and not before, as might be expected if homoplasy were a major contributor to pattern predictions (Fig 3). In addition, one of the oldest acquired mutations toward XDR-level resistance was a specific 130 bp deletion in *gidB*, which is extremely unlikely to arise many times independently and supports accurate reconstruction in our evolutionary analysis. Furthermore, though our calculated mutation rate for LAM4 was slightly higher than previous reports [39,41,46–48], our estimate was within the reported 95% HPD interval and was based on a larger fraction of the H37Rv genome than previous studies (99.9% versus <90% H37Rv mapping coverage) [39]. This was due to the inclusion of sequence data generated from both PCR-free short fragment and jumping libraries and analysis with improved bioinformatics tools that enabled us to examine SNPs within more variable and high guanine-cytosine (GC) content regions of the genome, including proline-glutamic acid (PE) and proline-proline-glutamic acid (PPE) genes that have been reported to have a higher mutation rate [58]. Thus, because we are including data from more of the genome, our estimation of the *M*. *tuberculosis* mutation rate may more closely approximate the actual mutation rate of the organism as compared to previously published studies.

Importantly, from the pattern of drug resistance evolution within LAM4, it is clear that the precursors to XDR evolved well before the explosive South African HIV epidemic of the 1990s, indicating that the selection of transmissible XDR strains can occur in low-prevalence HIV settings. While recent failures in TB and infection control and the current high HIV prevalence rates, combined, undoubtedly contributed to the spread of XDR, they were not the sole causes of XDR in this setting. Indeed, strains that evolved first-line drug resistance soon after the introduction of chemotherapy were a critical entry point to today’s drug-resistant epidemic. Drug-resistant strains that emerged from the mid-20th century were evidently maintained within the population of *M*. *tuberculosis*, presenting the opportunity for the acquisition of successive resistance and compensatory mutations that culminated in transmissible XDR and the Tugela Ferry outbreak. Drug-resistant strains may have been maintained within a population over time either by ongoing cycles of infection and transmission or through reactivation of latent disease. It is unclear which of these may have been the most important in this setting, but it suggests that fitness costs due to first-line drug resistance may not be severe. Reactivation was recently shown to be important in the transcontinental spread of MDR-TB from Thailand to California over a 22-y period [59], but it is unclear whether this factor was also critical in KwaZulu-Natal.

Beyond LAM4, and as has been shown in other studies [10,14], drug resistance emerged de novo repeatedly in KwaZulu-Natal, as evidenced by our identification of numerous independent evolutionary events of MDR and XDR across multiple lineages and spoligotypes. Of particular note was the detection of multiple independent evolutions of MDR to pre-XDR within LAM4, which may herald a new wave of XDR in the near future. Thus, the repeated emergence of de novo high-level drug resistance underscores the reality that, even in middle-income sub-Saharan African countries, the current approach to TB control is failing to stem the ongoing emergence of drug resistance. In fact, results from our analyses suggest this was not due to infrequent poor adherence to TB drugs but instead to decades of inadequate TB control that has driven resistance development in a stepwise fashion, multiple times over. Given that our estimates of resistance evolution were based on identification of known resistance-conferring mutations and that the majority of sequenced strains were single colony purified, our calculations are likely an underestimation due to incomplete understanding of all mutations that confer drug resistance and the possibility of mixed infections, respectively. Thus, the state of drug resistance emergence is likely more dire than we have described.

Recent studies from KwaZulu-Natal have emphasized transmission of a limited number of strains as a driving force behind the emergence of drug resistance [10,14]. Our data also confirm that once drug resistance develops, clonal spread of resistant strains can and does occur in this context. We found that recent person-to-person spread of resistant strains is apparent in KwaZulu-Natal, as evidenced by identification of multiple drug-resistant clones. Importantly, in contrast to initial reports from Tugela Ferry in which nearly all XDR cases were TB/HIV coinfected [7,9], eight patients in our study in whom the Tugela Ferry XDR Clone was identified were HIV negative. This reemphasizes that even XDR drug-resistant strains are sufficiently fit to transmit person to person and cause morbidity in both immunocompetent and immunosuppressed persons. Improved infection control and rapid case finding will be necessary to prevent further spread of drug-resistant strains and to detect such cases in the community as well as in hospital settings [28].

Our genomic analysis uncovered a common initial pattern of drug resistance that is not optimally detected by current diagnostic algorithms. Isoniazid resistance was overwhelmingly the first drug resistance to occur along the pathway to multiple drug resistances. However, current TB control strategies in South Africa focus on early detection of rifampicin resistance as a surrogate marker of MDR and do not include the detection of isoniazid resistance. Clinical diagnostic policies that rely on Xpert MTB/RIF (a WHO-endorsed and widely deployed molecular diagnostic) [60] without more extensive drug resistance testing allow isoniazid resistance to go undetected and unchecked. Moreover, under current short course treatment guidelines that utilize 4 mo of isoniazid and rifampicin in the continuation phase [61], failure to recognize isoniazid monoresistance is tantamount to provision of unopposed rifampicin therapy and may rapidly select for rifampicin resistance. This phenomenon may be underappreciated and incompletely accounted for in mathematical models that recommend continued use of screening tools that identify only rifampicin resistance [62]. Furthermore, if rifampicin resistance is indeed detected by Xpert, failure to implement confirmatory secondary molecular testing for dual rifampin and isoniazid resistance, as is mandated by South African policy, occurs at unacceptably high rates [63]. Our ordering of drug resistance acquisition provides strong evidence that isoniazid monoresistance is a common pathway toward development of MDR and highlights the importance of prompt identification and treatment of isoniazid monoresistance. Failure to do so would be recapitulating the scenario that led to the current XDR problem.

Beyond detection, identification of the initial drivers of isoniazid monoresistance is also critical to the prevention of successive resistances. Isoniazid preventive therapy (IPT) has previously been implicated as a potential source of isoniazid monoresistance [64,65]. Our work highlights the need to understand the true risks of mass IPT implementation [66] in high-burden settings.

We were able to verify that the evolutionary patterns of select previously described rifampicin and isoniazid compensatory mutations do indeed appear to be consistent with compensation to their respective drug. Similarly, *ubiA* was observed to evolve in a stepping-stone pattern rather than a compensatory pattern with respect to ethambutol resistance [56]. Furthermore, we have identified novel putative rifampicin compensatory mutations that may have acted to restore bacterial fitness and facilitate transmission of drug-resistant strains. While the majority of the previously described rifampicin compensatory mutations had an evolutionary pattern consistent with this role, four polymorphisms previously associated with rifampicin compensation (*rpoB* I491F, *rpoC* G594E and N826K, and *rpoA* E319K) were not observed to evolve concurrently or subsequent to genotypic rifampicin resistance (S4 Table). These mutations may (i) not be compensatory mutations in the classic sense (i.e., mutations that evolve following gain of genotypic drug resistance to mitigate a fitness cost) but instead serve as stepping-stone mutations, (ii) evolve in concert with non-RRDR genotypic rifampicin resistance, or (iii) have no association with rifampicin resistance. We have proposed 26 novel mutations whose evolutionary patterns are consistent with rifampicin compensation, and these should be investigated in future studies.

The most commonly observed genotypic rifampicin resistance mutation among our sequenced strains was *rpoB* S450L (often referred to as S531L using the *Escherichia coli* codon numbering scheme), which is known to be the most prevalent RRDR mutation. Laboratory-derived strains carrying the S450L were previously shown to have relatively high fitness in in vitro growth assays [15], supporting the hypothesis that high prevalence of the S450L mutation among clinical strains was due to it imparting few fitness consequences. However, as shown in our study and in several others [20,54], *rpoB* S450L was the most likely RRDR polymorphism to evolve putative compensatory mutations, which calls into question the low fitness cost of S450L in vivo. Song et al. assessed rifampicin fitness by transcriptional efficiency (rather than growth) and showed that the S450L mutation has half the transcriptional efficiency of WT *rpoB* [54], which is likely to impart fitness consequences if not compensated.

This study has two main limitations. First, as our study isolates derived from only one geographic region, our conclusions regarding the timing and dating of the emergence of resistance may not be universal. However, two recent studies [67,68] have reported results compatible with ours from different settings. Using a similar approach, Eldholm et al. were able to date the first emergence of resistance in an MDR-TB outbreak from Argentina to the early 1970s and found that isoniazid and streptomycin resistance-conferring mutations were the first to be acquired. In a study of the global spread of the Beijing lineage [23], isoniazid and streptomycin resistances were also found to be common to all drug-resistant strains in two clonal complexes that resulted in the epidemic spread of two MDR clones in Russia and Central Asia 20 to 30 y ago. Another limitation, as discussed above, is that parsimony-based dating approaches may fail to distinguish rapid independent evolutions of a commonly occurring resistance mutation as two unique evolutionary events. This could lead to erroneous assignment of a mutation to a more basal part of the phylogenetic tree. While a theoretical risk, we believe the effect was minimal in our dataset since, as described above, our predictions were consistent with the timing of drug introduction, and we included a specific large deletion that is extremely unlikely to arise many times independently.

Here, we present the largest WGS study conducted to date of drug-resistant clinical isolates of *M*. *tuberculosis* from South Africa. Our dating analysis highlights the dire repercussions of failure to control first-line drug resistance. As acquisition of isoniazid resistance is the key initiation event for progression to MDR and beyond, TB control efforts that focus on the identification of isoniazid as well as rifampicin resistance will result in earlier detection of drug-resistant TB cases. Prudent antibiotic stewardship during the introduction of new antitubercular drugs will be critical to prevent the early fixation of resistance and protect the lifespan of novel agents.

## Supporting Information

S1 Fig Bootstrap values for phylogenetic tree of 337 strains (1,000 bootstrap replicates). (PDF)

S2 Fig Defining clones with varying SNP thresholds. The numbered columns to the right of the phylogenetic tree represent varying SNP thresholds used to define a clone. Strains that would be considered clonal by the SNP threshold listed in the column header are indicated by a unique three-letter code. By the ten-SNP threshold, the Tugela Ferry XDR Clone (labeled 10-AAI, shaded in gray) contains 50 members.
(PDF)

S3 Fig Drug-resistant clones are distributed widely across the phylogenetic tree. Columns to the right of the phylogenetic tree represent phenotypic DST (as indicated by the colored square), clones defined at the ten-SNP threshold as shown in S2 Table and S2 Fig, and the HIV status of sampled patient.
(PDF)

S4 Fig Bootstrap values for phylogenetic tree of LAM4 isolates shown in Fig 3 (1,000 bootstrap replicates). (PDF)

S1 Methods Microbiologic techniques; digital spoligotyping; clonal strain identification; genotypic definitions of drug resistance, accessory and compensatory mutations; and statistical tests. (DOCX)

S1 Table Participant data. Each participant was assigned a strain number with the header Tuberculosis KwaZulu-Natal K-RITH (TKK). Data for each participant included year of collection, specimen type, and smear status (if known). DNA isolation technique via single colony isolation (SCI) or non-single colony selection (non-SCI) is denoted. DST results are reported for each tested drug using the following abbreviations: rifampicin (R), isoniazid (H), nicotinamide (N), pyrazinamide (P), ethambutol (E), streptomycin (S), kanamycin (K), ofloxacin (O), ethionamide (Et), and capreomycin (C). DST results are noted as susceptible (S), resistant (R), or untested (U). Genomic spoligotyping and lineage that were derived from the sequencing data are listed. Lastly, genotypic drug susceptibility prediction and membership in the Tugela Ferry Clone are reported.
(PDF)

S2 Table Identification of drug-resistant clones indicates recent person-to-person transmission of drug-resistant TB. A linkage analysis identified 11 drug-resistant clones in the entire dataset. The largest clone contained 50 members of the LAM4 spoligotype; this spoligotype was subsequently identified as the Tugela Ferry XDR Clone. Within the LAM4 spoligotype, there were three additional clones identified, and clones were also identified in five other spoligotypes. All clone members were noted to be drug resistant, indicating recent person-to-person transmission of drug-resistant TB. See S1 Methods for definition of a clone.
(PDF)

S3 Table Diverse drug-resistant strains and frequent de novo development of drug resistance. Drug-resistant strains belonged to many distinct spoligotypes, which highlights the diversity of the drug resistance epidemic in this region. With a parsimony-based analysis, we quantified the independent evolutionary gains of genotypic MDR and XDR in our 340-strain dataset.
(PDF)

S4 Table Putative rifampicin compensatory mutations were identified in *rpoA*, *rpoC*, and non-RRDR regions of *rpoB*. Polymorphisms were deemed consistent with compensatory mutations when they evolved after or concurrent to genotypic rifampicin resistance. Many previously described putative compensatory mutations occurred in this evolutionary pattern, and 26 novel polymorphisms were newly described.
(XLSX)

S5 Table Distribution of putative rifampicin compensatory mutations across the RRDR. The vast majority of *rpoA*, *rpoC*, and non-RRDR *rpoB* mutations that evolved with an evolutionary pattern consistent with rifampicin compensation evolved in association with *rpoB* S450L.
(XLSX)

S6 Table Ordering of acquisition of polymorphisms with respect to genotypic resistance. *ahpC* and *ubiA* mutations were ordered with respect to genotypic isoniazid and ethambutol resistance, respectively.
(PDF)

## Abbreviations

BREC: Biomedical Research Ethics Council
CUBS: Collection of Urine Blood Sputum Study
DST: drug susceptibility testing
ESS: effective sample size
GC: guanine-cytosine
HPD: highest posterior density
IPT: isoniazid preventive therapy
KZNSUR: KwaZulu-Natal Drug Surveillance Study
MDR: multidrug resistant
NHLS: National Health Services Laboratory
PE: proline-glutamic acid
PPE: proline-proline-glutamic acid
PROX: Prospective Collection of Extensively Drug-Resistant TB
RRDR: rifampicin resistance-determining region
SCI: single colony isolation
SD: standard deviation
SNP: single nucleotide polymorphism
TB: tuberculosis
UNAIDS: The Joint United Nations Programme on HIV and AIDS
WGS: whole genome sequencing
XDR: extensively drug resistant
