# Supplementary figures and images for: Evolution of Extensively Drug-Resistant Tuberculosis over Four Decades: Whole Genome Sequencing and Dating Analysis of Mycobacterium tuberculosis Isolates from KwaZulu-Natal
Source: PLoS Med. 2015 Sep 29;12(9):e1001880. doi: 10.1371/journal.pmed.1001880 (PMC4587932; doi:10.1371/journal.pmed.1001880)

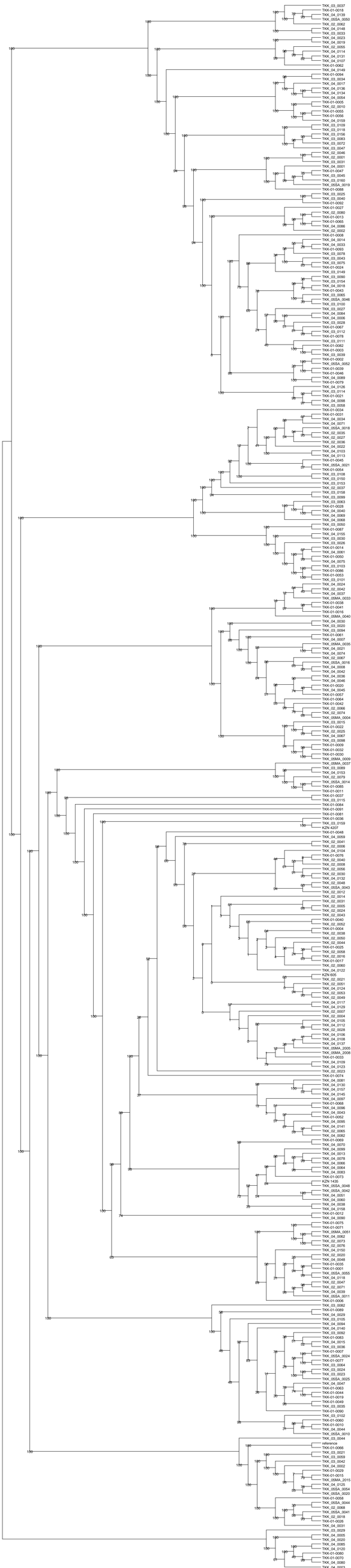

Supplement: S1 Fig — (PDF) [file pmed.1001880.s001.pdf]

12 15 20 25 30 35 40 45 50 100 200 500 1000 2000 3000

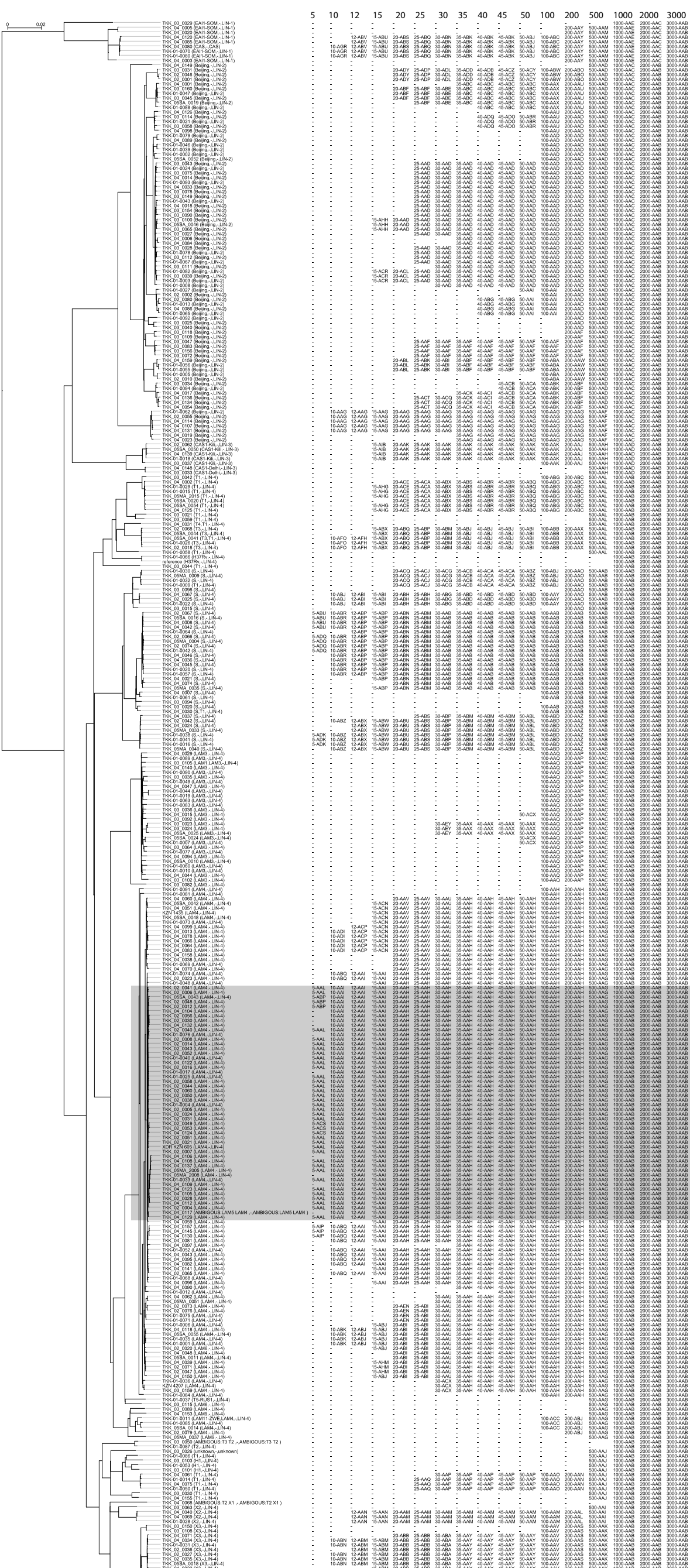

Supplement: S2 Fig — The numbered columns to the right of the phylogenetic tree represent varying SNP thresholds used to define a clone. Strains that would be considered clonal by the SNP threshold listed in the column header are indicated by a unique three-letter code. By the ten-SNP threshold, the Tugela Ferry XDR Clone (labeled 10-AAI, shaded in gray) contains 50 members. (PDF) [file pmed.1001880.s002.pdf]

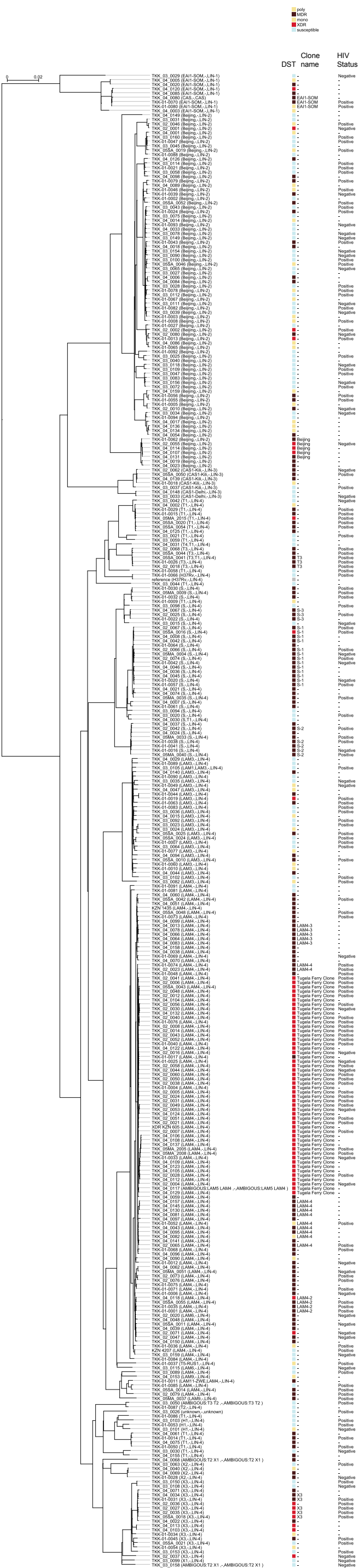

Supplement: S3 Fig — Columns to the right of the phylogenetic tree represent phenotypic DST (as indicated by the colored square), clones defined at the ten-SNP threshold as shown in S2 Table and S2 Fig, and the HIV status of sampled patient. (PDF) [file pmed.1001880.s003.pdf]

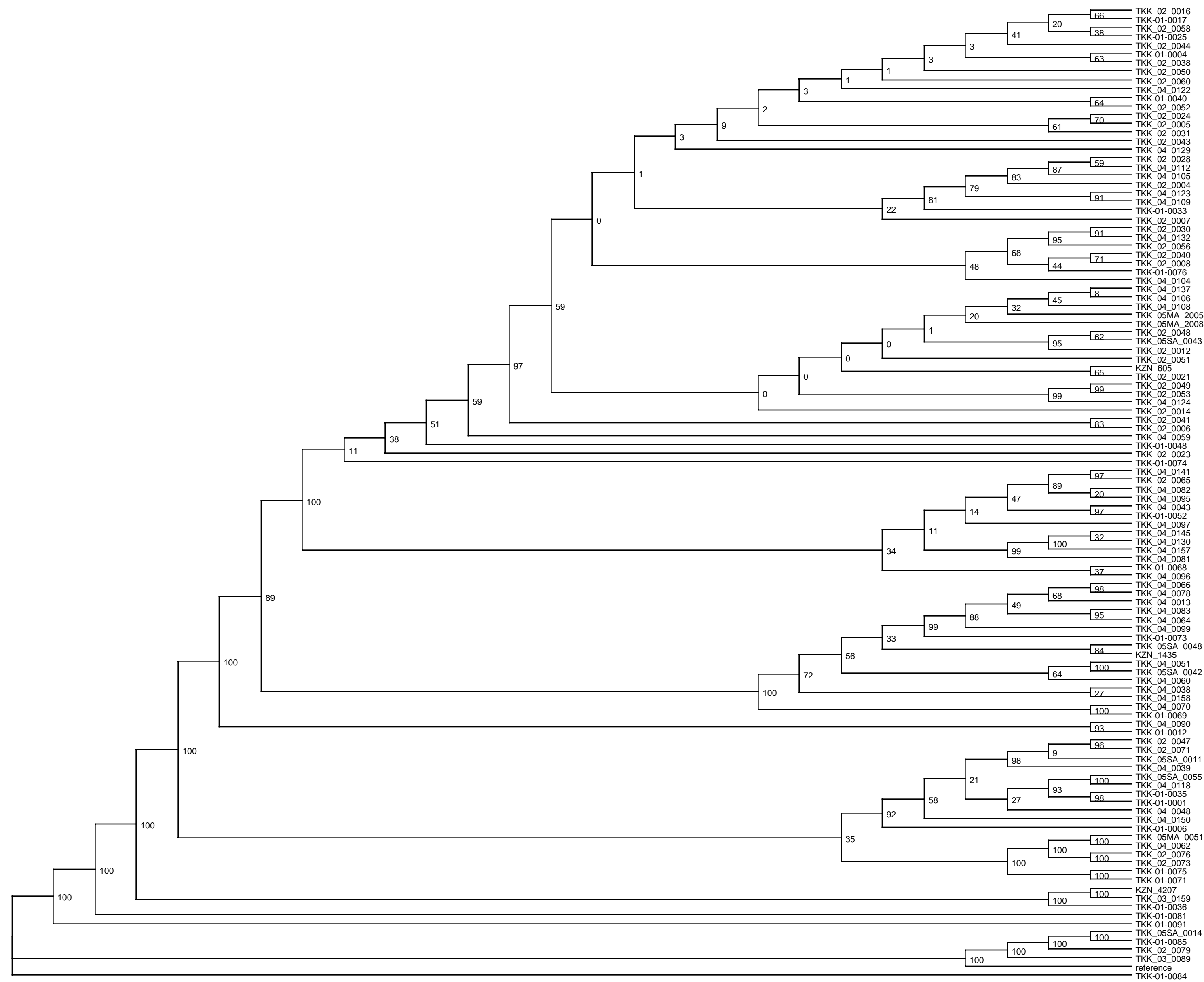

Supplement: S4 Fig — (PDF) [file pmed.1001880.s004.pdf]
