# Supplementary material for: Evolution of Extensively Drug-Resistant Tuberculosis over Four Decades: Whole Genome Sequencing and Dating Analysis of Mycobacterium tuberculosis Isolates from KwaZulu-Natal
Source: PLoS Med. 2015 Sep 29;12(9):e1001880. doi: 10.1371/journal.pmed.1001880 (PMC4587932; doi:10.1371/journal.pmed.1001880)
Supplement: S1 Methods — (DOCX) [file pmed.1001880.s005.docx]

**Supplemental Web Appendix**

**Supplemental text to “Evolution of extensively drug-resistant tuberculosis over four decades: whole genome sequencing and dating analysis of Mycobacterium tuberculosis isolates from**

**KwaZulu-Natal”**

**Authors:**

Keira A. Cohen^1,2‡^, Thomas Abeel^3,4‡^, Abigail Manson McGuire^3^, Christopher A. Desjardins^3^, Vanisha Munsamy^2^, Terrance P. Shea^3^, Bruce J. Walker^3^, Nonkqubela Bantubani^5^, Deepak V. Almeida^2,11^, Lucia Alvarado^3^, Sinéad B. Chapman^3^, Nomonde R. Mvelase^6,7^, Eamon Y. Duffy^2^, Michael G. Fitzgerald^3^, Pamla Govender^2^, Sharvari Gujja^3^, Susanna Hamilton^3^, Clinton Howarth^3^, Jeffrey D. Larimer^3^, Kashmeel Maharaj^2^, Matthew D. Pearson^3^, Margaret E. Priest^3^, Qiandong Zeng^3^, Nesri Padayatchi^8^, Jacques Grosset^2,11^, Sarah K. Young^3^, Jennifer Wortman^3^, Koleka P. Mlisana^6,7^, Max R. O'Donnell^8,9,10^, Bruce W. Birren^3^, William R. Bishai^11^, Alexander S. Pym^2*†^ and Ashlee M. Earl ^3*†^

**Affiliations:**

1. Division of Pulmonary and Critical Care Medicine, Brigham and Women’s Hospital, Harvard Medical School, Boston, MA, USA

2. KwaZulu-Natal Research Institute for TB and HIV (K-RITH), Durban, South Africa

3. Broad Institute of MIT and Harvard, Cambridge, MA, USA

4. Delft Bioinformatics Lab, Delft University of Technology, Delft, The Netherlands

5. Medical Research Council, Durban, South Africa

6. School of Laboratory Medicine and Medical Sciences, University of KwaZulu-Natal, Durban, South Africa

7. National Health Laboratory Service, Durban, South Africa

8. Centre for the AIDS Programme of Research in South Africa (CAPRISA), Durban, South Africa

9. Division of Pulmonary, Allergy, and Critical Care Medicine, Columbia University College of Physicians and Surgeons, New York, USA

10. Department of Epidemiology, Columbia Mailman School of Public Health, New York, USA

11. Center for Tuberculosis Research, Johns Hopkins School of Medicine, Baltimore, MD, USA

^*^These authors contributed equally

^†^These authors also contributed equally

**Appendix: Supplemental Methods**

**Drug Susceptibility Testing**

Phenotypic DST was performed prospectively by indirect proportion method on Middlebrook 7H10 agar plates supplemented with the drug concentrations listed in Table S2.

**Storage and recovery of retrospectively collected strains**

At the initial time of specimen collection of the retrospective cohorts, sputum specimens were directly cultured on solid agar after sputum decontamination with N-acetylcysteine. Drug susceptibility testing was performed prospectively. Cultures were subsequently banked in glycerol and stored at -80°C. To recover the freezer stocks for use in this study, a loopful of the frozen glycerol stock was inoculated onto Middlebrook 7H10 plates in a four-way streak and incubated at 37°C.

**Single Colony Selection**

As indicated in appendix table 1, a subset of isolates underwent single colony selection prior to DNA extraction. To accomplish this selection, a single colony was selected from an agar 7H10 or 7H11 plate and inoculated into a 50mL conical tube containing 10mL of Middlebrook 7H9 media. The resulting inoculum was incubated at 37°C in a shaking incubator and monitored until the culture achieved late log phase growth (OD_600_ of approximately 0.8 to 1.0).

**Genomic DNA extraction**

Extraction of genomic DNA was performed on 10mL cultures grown in 7H9 broth using the CTAB-lysozyme method as previously described [1].

**Digital** **Spoligotyping**

Spoligotyping, or spacer oligotyping, is a molecular typing tool that interrogates the direct repeat (DR) locus and adjacent spacer sequences of *M. tb* to differentiate and group taxons of strains [2]. In wet lab spoligotyping, the CRISPR region is PCR amplified, and the resulting DNA is hybridized to a membrane where the presence or absence of 43 unique spacer sequences can be visualized. We developed a digital method for spoligotyping that was based on sequenced reads. In this approach, reads from each isolate were matched against known spacer marker sequences. Matching reads were totaled to determine if markers were present or absent. For each isolate, we assessed the presence (or absence) of 43 spacer sequences that are traditionally used for spoligotyping [2]. Markers were considered absent if the read count totals were in the lowest quartile of counts. The count totals for absent markers were subsequently used to build a background model for the presence test. Present markers were determined by calculating a Bonferroni corrected p-value based on an exponential distribution with average counts of the absent markers. Markers with a p-value < 0.01 were considered to be present. Results were matched to spacer pattern profiles at SITVITWEB to generate a named spoligotype for each isolate [3].

**Clonal Strain Identification**

Strains were grouped in clusters using a DBSCAN based algorithm [4]. We used an epsilon range query of ten SNPs and a minimum core cluster size of three strains. As a result, only groups of at least three or more strains were clustered into clonal groups. We used ten SNP differences as a maximum threshold for inclusion in a clone since number was in line with the numbers of SNPs observed between strains isolated in established transmission chains [5,6]. As expected, varying the SNP threshold that defines a clone changed the number of clones identified in our dataset.

**Genotypic Drug Resistance Definitions**

A curated list of genomic polymorphisms that confer drug resistance was defined for each tested drug based on a literature review and consideration of the components of current molecular drug resistance diagnostics. Resistance mutations were selected for inclusion based on conservative criteria with only laboratory-proven resistance polymorphisms and mechanisms included. As a first pass, all mutations incorporated in current molecular diagnostics in standard practice were included. This included the Xpert MTB/RIF[7][8], the Hain Genotype MTBDR*plus* [9] and the Hain Genotype MTBDR*sl* Line Probe Assay [10]. Select additional mutations were added if there was sufficient laboratory evidence to include them.

**Rifampicin**: All *rpoB* mutations incorporated in the Xpert or Hain MTBDR*plus* were included. This resistance definition was expanded to include all non-synonymous mutations in the 81 base pair core of *rpoB* [11], which has previously been termed the Rifampin Resistance Determining Region (RRDR).

**Isoniazid**: The *katG* S315 mutation and the *inhA* promoter polymorphisms incorporated in the Hain MTBDR*plus* were included*.* This resistance definition was expanded to include loss of function (frameshift or nonsense) mutations of *katG* [12].

**Ethambutol:** The non-synonymous *embB* M306 mutation included on the Hain Genotype MTBDR*sl* was added. This resistance definition was expanded to include two additional *embB* codons—G406 and Q497—which have previously been named as part of the Ethambutol Resistance Determining Region (ERDR) [13].

**Pyrazinamide:** Genotypic resistance was defined as any loss of function (frameshift or nonsense) mutation in the *pncA* gene [14] or in the *pncA* promoter [15].

**Fluoroquinolones:** The non-synonymous *gyrA* mutations incorporated as part of the Hain Genotype MTBDR*sl—agyrA* codons A90, S91 and D94--were included. This resistance definition was expanded to include an additional codon of *gyrA*, G88, which is considered part of the Quinolone Resistance Determining Region (QRDR) of *gyrA.*[16] Non-synonymous mutations of the *gyrB* QRDR were also included [16].

**Streptomycin:** Genotypic resistance to streptomycin was defined by mutations in the *rrs* streptomycin-resistance determining positions (*rrs* 513, 516, 906 and 907) and *rpsL* codons K43 and K88 [17–20]. Interruption of a third gene, *gidB*, has also been shown to confer low-level resistance to streptomycin [21], so loss of function (frameshift and nonsense) mutations in *gidB* were also considered indicative of genotypic streptomycin resistance.

**Kanamycin:** The *rrs* 1400 mutation included on the Hain MTBDR*sl* was considered to confer kanamycin resistance*.* Additional resistance mutations included those at *rrs* 1401 and 1483 [22] as well as in the *eis* promoter [22].

**Ethionamide:** Genotypic resistance to ethionamide was conservatively defined as loss of function (frameshift or nonsense) mutations in *ethA* [23]*.*

**Accessory/Compensatory Mutation Definitions**

A list of genomic polymorphisms putatively involved in compensating for acquired resistance mutations was developed for rifampicin and isoniazid. For ethambutol we defined accessory mutations that have been shown to increase the MIC to ethambutol.

**Rifampicin:** The *rpoA, rpoC* and non-RRDR *rpoB* polymorphisms that were identified in previous studies were included.[24–27]

**Isoniazid:** Compensatory mutations for isoniazid resistance were defined genotypically as mutations that occurred in the *ahpC* promoter [28,29].

**Ethambutol:** Accessory mutations for ethambutol resistance were defined as non-synonymous mutations in Rv3806c (*ubiA*), which has been shown to double the ethambutol MIC for clinical isolates of *M. tb* [30].

**Statistical tests**

Categorical variable variables were compared by Fisher’s exact test and continuous variables by non-parametric Mann-Whitney test. A p value < 0.05 was considered significant. Statistical analyses were performed with Prism GraphPad version 6.0. (GraphPad, San Diego, CA)

**Supplemental Web Appendix References**

1. Larsen MH, Biermann K, Tandberg S, Hsu T, Jacobs WR. Genetic Manipulation of Mycobacterium tuberculosis. Curr Protoc Microbiol. 2007;Chapter 10: Unit 10A.2. doi:10.1002/9780471729259.mc10a02s6

2. Kamerbeek J, Schouls L, Kolk a, van Agterveld M, van Soolingen D, Kuijper S, et al. Simultaneous detection and strain differentiation of Mycobacterium tuberculosis for diagnosis and epidemiology. J Clin Microbiol. 1997;35: 907–14.

3. Demay C, Liens B, Burguière T, Hill V, Couvin D, Millet J, et al. SITVITWEB--a publicly available international multimarker database for studying Mycobacterium tuberculosis genetic diversity and molecular epidemiology. Infect Genet Evol. 2012;12: 755–66. doi:10.1016/j.meegid.2012.02.004

4. Ester M, Kriegel H-P, Jörg S, Xu X. A density-based algorithm for discovering clusters in large spatial databases with noise. In: Simoudis, Evangelos; Han, Jiawei; Fayyad UM, editor. Proceedings of the Second International Conference on Knowledge Discovery and Data Mining (KDD-96). AAAI Press; 1996. pp. 226–231. doi:10.1.1.71.1980

5. Walker TM, Lalor MK, Broda A, Saldana Ortega L, Morgan M, Parker L, et al. Assessment of Mycobacterium tuberculosis transmission in Oxfordshire, UK, 2007-12, with whole pathogen genome sequences: an observational study. Lancet Respir Med. 2014;2: 285–92. doi:10.1016/S2213-2600(14)70027-X

6. Walker TM, Ip CL, Harrell RH, Evans JT, Kapatai G, Dedicoat MJ, et al. Whole-genome sequencing to delineate Mycobacterium tuberculosis outbreaks: a retrospective observational study. Lancet Infect Dis. Elsevier Ltd; 2013;13: 137–46. doi:10.1016/S1473-3099(12)70277-3

7. Boehme CC, Nabeta P, Hillemann D, Nicol MP, Shenai S, Krapp F, et al. Rapid molecular detection of tuberculosis and rifampin resistance. N Engl J Med. 2010;363: 1005–15. doi:10.1056/NEJMoa0907847

8. Xpert MTB/RIF [Internet]. Available: http://www.cepheid.com/us/cepheid-solutions/clinical-ivd-tests/critical-infectious-diseases/xpert-mtb-rif

9. Hain Genotype MTBDRplus [Internet]. Available: http://www.hain-lifescience.de/en/products/microbiology/mycobacteria/genotype-mtbdrplus.html

10. Hain Genotype MTBDRsl [Internet]. Available: http://www.hain-lifescience.de/en/products/microbiology/mycobacteria/genotype-mtbdrsl.html

11. Telenti A, Imboden P, Marchesi F, Lowrie D, Cole S, Colston MJ, et al. Detection of rifampicin-resistance mutations in Mycobacterium tuberculosis. Lancet. 1993;341: 647–50.

12. Heym B, Alzari PM, Honoré N, Cole ST. Missense mutations in the catalase-peroxidase gene, katG, are associated with isoniazid resistance in Mycobacterium tuberculosis. Mol Microbiol. 1995;15: 235–45.

13. Alcaide F, Pfyffer GE, Telenti A. Role of embB in natural and acquired resistance to ethambutol in mycobacteria. Antimicrob Agents Chemother. 1997;41: 2270–3.

14. Scorpio A, Zhang Y. Mutations in pncA, a gene encoding pyrazinamidase/nicotinamidase, cause resistance to the antituberculous drug pyrazinamide in tubercle bacillus. Nat Med. 1996;2: 662–7.

15. Sreevatsan S, Pan X, Zhang Y, Kreiswirth BN, Musser JM. Mutations associated with pyrazinamide resistance in pncA of Mycobacterium tuberculosis complex organisms. Antimicrob Agents Chemother. 1997;41: 636–40.

16. Maruri F, Sterling TR, Kaiga AW, Blackman A, van der Heijden YF, Mayer C, et al. A systematic review of gyrase mutations associated with fluoroquinolone-resistant Mycobacterium tuberculosis and a proposed gyrase numbering system. J Antimicrob Chemother. 2012;67: 819–31. doi:10.1093/jac/dkr566

17. Finken M, Kirschner P, Meier A, Wrede A, Böttger EC. Molecular basis of streptomycin resistance in Mycobacterium tuberculosis: alterations of the ribosomal protein S12 gene and point mutations within a functional 16S ribosomal RNA pseudoknot. Mol Microbiol. 1993;9: 1239–46.

18. Honoré N, Cole ST. Streptomycin resistance in mycobacteria. Antimicrob Agents Chemother. 1994;38: 238–42.

19. Nair J, Rouse D a, Bai GH, Morris SL. The rpsL gene and streptomycin resistance in single and multiple drug-resistant strains of Mycobacterium tuberculosis. Mol Microbiol. 1993;10: 521–7.

20. Meier A, Kirschner P, Bange FC, Vogel U, Böttger EC. Genetic alterations in streptomycin-resistant Mycobacterium tuberculosis: mapping of mutations conferring resistance. Antimicrob Agents Chemother. 1994;38: 228–33. doi:10.1128/AAC.38.2.228.Updated

21. Wong SY, Lee JS, Kwak HK, Via LE, Boshoff HIM, Barry CE. Mutations in gidB confer low-level streptomycin resistance in Mycobacterium tuberculosis. Antimicrob Agents Chemother. 2011;55: 2515–22. doi:10.1128/AAC.01814-10

22. Suzuki Y, Katsukawa C, Tamaru A, Abe C, Makino M, Mizuguchi Y, et al. Detection of kanamycin-resistant Mycobacterium tuberculosis by identifying mutations in the 16S rRNA gene. J Clin Microbiol. 1998;36: 1220–5.

23. Morlock GP, Metchock B, Sikes D, Crawford JT, Cooksey RC. ethA, inhA, and katG loci of ethionamide-resistant clinical Mycobacterium tuberculosis isolates. Antimicrob Agents Chemother. 2003;47: 3799–805. doi:10.1128/AAC.47.12.3799

24. Comas I, Borrell S, Roetzer A, Rose G, Malla B, Kato-Maeda M, et al. Whole-genome sequencing of rifampicin-resistant Mycobacterium tuberculosis strains identifies compensatory mutations in RNA polymerase genes. Nat Genet. Nature Publishing Group; 2012;44: 106–10. doi:10.1038/ng.1038

25. Casali N, Nikolayevskyy V, Balabanova Y, Harris SR, Ignatyeva O, Kontsevaya I, et al. Evolution and transmission of drug-resistant tuberculosis in a Russian population. Nat Genet. Nature Publishing Group; 2014;46: 279–86. doi:10.1038/ng.2878

26. De Vos M, Müller B, Borrell S, Black P a., Van Helden PD, Warren RM, et al. Putative compensatory mutations in the rpoc gene of rifampin-resistant mycobacterium tuberculosis are associated with ongoing transmission. Antimicrob Agents Chemother. 2013;57: 827–832. doi:10.1128/AAC.01541-12

27. Song T, Park Y, Shamputa IC, Seo S, Lee SY, Jeon HS, et al. Fitness costs of rifampicin resistance in Mycobacterium tuberculosis are amplified under conditions of nutrient starvation and compensated by mutation in the β′ subunit of RNA polymerase. Mol Microbiol. 2014;91: 1106–1119. doi:10.1111/mmi.12520

28. Wilson TM, Collins DM. ahpC, a gene involved in isoniazid resistance of the Mycobacterium tuberculosis complex. Mol Microbiol. 1996;19: 1025–34.

29. Kelley CL, Rouse DA, Morris SL. Analysis of ahpC gene mutations in isoniazid-resistant clinical isolates of Mycobacterium tuberculosis. Antimicrob Agents Chemother. 1997;41: 2057–8.

30. Safi H, Lingaraju S, Amin A, Kim S, Jones M, Holmes M, et al. Evolution of high-level ethambutol-resistant tuberculosis through interacting mutations in decaprenylphosphoryl-β-D-arabinose biosynthetic and utilization pathway genes. Nat Genet. Nature Publishing Group; 2013;45: 1190–7. doi:10.1038/ng.2743
