# Supplementary material for: Evolution of Extensively Drug-Resistant Tuberculosis over Four Decades: Whole Genome Sequencing and Dating Analysis of Mycobacterium tuberculosis Isolates from KwaZulu-Natal
Source: PLoS Med. 2015 Sep 29;12(9):e1001880. doi: 10.1371/journal.pmed.1001880 (PMC4587932; doi:10.1371/journal.pmed.1001880)
Supplement: S1 Table — Each participant was assigned a strain number with the header Tuberculosis KwaZulu-Natal K-RITH (TKK). Data for each participant included year of collection, specimen type, and smear status (if known). DNA isolation technique via single colony isolation (SCI) or non-single colony selection (non-SCI) is denoted. DST results are reported for each tested drug using the following abbreviations: rifampicin (R), isoniazid (H), nicotinamide (N), pyrazinamide (P), ethambutol (E), streptomycin (S), kanamycin (K), ofloxacin (O), ethionamide (Et), and capreomycin (C). DST results are noted as susceptible (S), resistant (R), or untested (U). Genomic spoligotyping and lineage that were derived from the sequencing data are listed. Lastly, genotypic drug susceptibility prediction and membership in the Tugela Ferry Clone are reported. (PDF) [file pmed.1001880.s006.pdf]

**Specimen ID**	**Cohort**	**Year**	**Specimen Type**	**Smear Status**	**DNA isolation**	**Phenotypic DST Pattern**	**C**	**E**	**Et**	**H**	**K**	**P/N**	**O**	**R**	**S**	**Digital Spoligotype**	**Lineage**	**Genotypic DST pattern**	**Tugela Ferry vs. non-Tugela Ferry XDR**
**TKK-01-0001**	KZNSUR	2008	sputum	Negative	SCI	MDR	S	R	R	R	S	R	S	R	R	LAM4	LIN 4	MDR	n/a
**TKK-01-0002**	KZNSUR	2008	sputum	Positive	SCI	susceptible	S	S	S	S	S	S	S	S	S	Beijing	LIN 2	susceptible	n/a
**TKK-01-0003**	KZNSUR	2008	sputum	Negative	SCI	mono	S	S	S	S	S	S	S	R	S	Beijing	LIN 2	susceptible	n/a
**TKK-01-0004**	KZNSUR	2008	sputum	Positive	SCI	XDR	R	R	S	R	R	R	R	R	R	LAM4	LIN 4	XDR	Tugela Ferry XDR
**TKK-01-0005**	KZNSUR	2008	sputum	Negative	SCI	poly	S	S	R	R	S	S	S	S	R	Beijing	LIN 2	Drug-resistant (other)	n/a
**TKK-01-0006**	KZNSUR	2008	sputum	Positive	SCI	MDR	S	S	S	R	S	S	S	R	S	LAM4	LIN 4	MDR	n/a
**TKK-01-0007**	KZNSUR	2008	sputum	Negative	SCI	susceptible	S	S	S	S	S	S	S	S	S	LAM3	LIN 4	susceptible	n/a
**TKK-01-0008**	KZNSUR	2008	sputum	Negative	SCI	mono	S	S	S	S	S	S	S	R	S	Beijing	LIN 2	Drug-resistant (other)	n/a
**TKK-01-0009**	KZNSUR	2008	sputum	Positive	SCI	mono	S	S	S	S	S	S	S	R	S	T1	LIN 4	MDR	n/a
**TKK-01-0010**	KZNSUR	2008	sputum	Positive	SCI	poly	S	S	S	R	S	S	S	S	R	LAM3	LIN 4	Drug-resistant (other)	n/a
**TKK-01-0011**	KZNSUR	2008	sputum	Positive	SCI	MDR	S	S	S	R	S	S	S	R	S	LAM11-ZWE,LAM4	LIN 4	MDR	n/a
**TKK-01-0012**	KZNSUR	2008	sputum	Positive	SCI	MDR	S	R	S	R	S	R	S	R	R	LAM4	LIN 4	MDR	n/a
**TKK-01-0013**	KZNSUR	2008	sputum	Positive	SCI	XDR	S	R	S	R	R	R	R	R	R	Beijing	LIN 2	XDR	non-Tugela Ferry XDR
**TKK-01-0014**	KZNSUR	2008	sputum	Positive	SCI	MDR	S	R	S	R	S	R	S	R	R	T1	LIN 4	MDR	n/a
**TKK-01-0015**	KZNSUR	2008	sputum	Positive	SCI	MDR	R	S	S	R	R	S	S	R	R	T1	LIN 4	MDR	n/a
**TKK-01-0016**	KZNSUR	2008	sputum	Positive	SCI	MDR	S	R	R	R	S	S	S	R	R	S	LIN 4	MDR	n/a
**TKK-01-0017**	KZNSUR	2008	sputum	Negative	SCI	MDR	R	R	R	R	R	R	S	R	R	LAM4	LIN 4	MDR	n/a
**TKK-01-0018**	KZNSUR	2008	sputum	Positive	SCI	poly	S	S	S	R	S	R	S	S	R	CAS1-Kili	LIN 3	Drug-resistant (other)	n/a
**TKK-01-0019**	KZNSUR	2008	sputum	Negative	SCI	XDR	S	R	R	R	R	R	R	R	R	LAM3	LIN 4	XDR	non-Tugela Ferry XDR
**TKK-01-0020**	KZNSUR	2008	sputum	Positive	SCI	MDR	S	S	S	R	S	S	S	R	R	S	LIN 4	MDR	n/a
**TKK-01-0021**	KZNSUR	2008	sputum	Positive	SCI	susceptible	S	S	S	S	S	S	S	S	S	Beijing	LIN 2	susceptible	n/a
**TKK-01-0022**	KZNSUR	2008	sputum	Positive	SCI	MDR	S	S	S	R	S	R	R	R	S	S	LIN 4	MDR	n/a
**TKK-01-0024**	KZNSUR	2008	sputum	Positive	SCI	MDR	S	S	S	R	S	S	S	R	S	Beijing	LIN 2	Drug-resistant (other)	n/a
**TKK-01-0025**	KZNSUR	2008	sputum	Positive	SCI	XDR	R	R	R	R	R	R	R	R	R	LAM4	LIN 4	XDR	Tugela Ferry XDR
**TKK-01-0026**	KZNSUR	2008	sputum	Positive	SCI	MDR	S	S	S	R	S	S	S	R	R	T3	LIN 4	MDR	n/a
**TKK-01-0027**	KZNSUR	2008	sputum	Positive	SCI	susceptible	S	S	S	S	S	S	S	S	S	Beijing	LIN 2	susceptible	n/a
**TKK-01-0028**	KZNSUR	2008	sputum	Positive	SCI	MDR	S	S	S	R	S	S	S	R	R	X2	LIN 4	MDR	n/a

Specimen ID	Cohort	Year	Specimen Type	Smear Status	DNA isolation	Phenotypic DST Pattern	C	E	Et	H	K	P/N	O	R	S	Digital Spoligotype	Lineage	Genotypic DST pattern	Tugela Ferry vs. non-Tugela Ferry XDR
**TKK-01-0029**	KZNSUR	2008	sputum	Negative	SCI	MDR	S	S	S	R	S	S	S	R	R	T1	LIN 4	MDR	n/a
**TKK-01-0030**	KZNSUR	2008	sputum	Positive	SCI	MDR	S	S	S	R	S	S	S	R	S	S	LIN 4	MDR	n/a
**TKK-01-0031**	KZNSUR	2008	sputum	Positive	SCI	MDR	S	S	S	R	S	S	S	R	S	X3	LIN 4	MDR	n/a
**TKK-01-0032**	KZNSUR	2008	sputum	Positive	SCI	MDR	S	S	S	R	S	S	S	R	S	S	LIN 4	MDR	n/a
**TKK-01-0033**	KZNSUR	2008	sputum	Positive	SCI	XDR	R	R	R	R	R	R	R	R	R	LAM4	LIN 4	XDR	Tugela Ferry XDR
**TKK-01-0034**	KZNSUR	2008	sputum	Positive	SCI	poly	S	S	S	R	S	S	S	S	R	X3	LIN 4	Drug-resistant (other)	n/a
**TKK-01-0035**	KZNSUR	2008	sputum	Positive	SCI	MDR	S	R	S	R	S	R	S	R	R	LAM4	LIN 4	MDR	n/a
**TKK-01-0036**	KZNSUR	2008	sputum	Positive	SCI	mono	S	S	S	S	S	S	S	S	R	LAM4	LIN 4	Drug-resistant (other)	n/a
**TKK-01-0037**	KZNSUR	2008	sputum	Negative	SCI	mono	S	S	S	R	S	S	S	S	S	T5-RUS1	LIN 4	Drug-resistant (other)	n/a
**TKK-01-0038**	KZNSUR	2008	sputum	unknown	SCI	MDR	S	R	R	R	S	S	S	R	R	S	LIN 4	MDR	n/a
**TKK-01-0039**	KZNSUR	2008	sputum	Positive	SCI	MDR	S	S	S	R	S	S	S	R	R	Beijing	LIN 2	MDR	n/a
**TKK-01-0040**	KZNSUR	2008	sputum	Positive	SCI	XDR	R	R	R	R	R	R	R	R	R	LAM4	LIN 4	XDR	Tugela Ferry XDR
**TKK-01-0041**	KZNSUR	unknown	sputum	unknown	SCI	MDR	R	R	R	R	S	S	S	R	R	S	LIN 4	MDR	n/a
**TKK-01-0042**	KZNSUR	2008	sputum	Positive	SCI	MDR	S	R	S	R	S	S	S	R	R	S	LIN 4	MDR	n/a
**TKK-01-0043**	KZNSUR	2008	sputum	Positive	SCI	MDR	S	R	S	R	S	S	S	R	R	Beijing	LIN 2	MDR	n/a
**TKK-01-0044**	KZNSUR	2008	sputum	Positive	SCI	MDR	S	S	S	R	S	S	S	R	S	LAM3	LIN 4	MDR	n/a
**TKK-01-0045**	KZNSUR	2008	sputum	Positive	SCI	MDR	S	S	S	R	S	S	S	R	R	X3	LIN 4	MDR	n/a
**TKK-01-0046**	KZNSUR	2008	sputum	Positive	SCI	poly	S	S	S	R	S	S	S	S	R	Beijing	LIN 2	Drug-resistant (other)	n/a
**TKK-01-0047**	KZNSUR	2008	sputum	Negative	SCI	susceptible	S	S	S	S	S	S	S	S	S	Beijing	LIN 2	susceptible	n/a
**TKK-01-0048**	KZNSUR	2009	sputum	Positive	SCI	MDR	S	R	R	R	S	R	S	R	R	LAM4	LIN 4	MDR	n/a
**TKK-01-0049**	KZNSUR	2009	sputum	Negative	SCI	mono	S	S	S	R	S	S	S	S	S	LAM3	LIN 4	Drug-resistant (other)	n/a
**TKK-01-0050**	KZNSUR	2009	sputum	Negative	SCI	MDR	S	R	R	R	S	R	S	R	S	T1	LIN 4	MDR	n/a
**TKK-01-0052**	KZNSUR	2009	sputum	Positive	SCI	poly	S	S	R	S	R	S	S	S	S	LAM4	LIN 4	MDR	n/a
**TKK-01-0053**	KZNSUR	2009	sputum	Negative	SCI	susceptible	S	S	S	S	S	S	S	S	S	H1	LIN 4	Drug-resistant (other)	n/a
**TKK-01-0054**	KZNSUR	2009	sputum	Positive	SCI	mono	S	S	S	S	S	R	S	S	S	X3	LIN 4	susceptible	n/a
**TKK-01-0055**	KZNSUR	2009	sputum	Negative	SCI	MDR	S	S	S	R	S	S	S	R	S	Beijing	LIN 2	MDR	n/a
**TKK-01-0056**	KZNSUR	2009	sputum	Negative	SCI	MDR	R	S	S	R	S	S	S	R	S	Beijing	LIN 2	MDR	n/a

Specimen ID	Cohort	Year	Specimen Type	Smear Status	DNA isolation	Phenotypic DST Pattern	C	E	Et	H	K	P/N	O	R	S	Digital Spoligotype	Lineage	Genotypic DST pattern	Tugela Ferry vs. non-Tugela Ferry XDR
**TKK-01-0057**	KZNSUR	2009	sputum	Negative	SCI	MDR	S	R	S	R	S	R	S	R	R	S	LIN 4	MDR	n/a
**TKK-01-0058**	KZNSUR	2009	sputum	Positive	SCI	susceptible	S	S	S	S	S	S	S	S	S	T1	LIN 4	susceptible	n/a
**TKK-01-0060**	KZNSUR	2010	sputum	Negative	SCI	poly	R	S	S	R	S	S	S	S	S	LAM3	LIN 4	Drug-resistant (other)	n/a
**TKK-01-0061**	KZNSUR	2010	sputum	Negative	SCI	MDR	S	R	S	R	S	S	S	R	R	S	LIN 4	MDR	n/a
**TKK-01-0062**	KZNSUR	2010	sputum	Negative	SCI	MDR	R	R	R	R	R	R	S	R	R	Beijing	LIN 2	MDR	n/a
**TKK-01-0063**	KZNSUR	2010	sputum	Negative	SCI	MDR	S	S	S	R	R	S	S	R	S	LAM3	LIN 4	MDR	n/a
**TKK-01-0064**	KZNSUR	2010	sputum	Positive	SCI	MDR	S	R	S	R	S	R	S	R	R	S	LIN 4	MDR	n/a
**TKK-01-0065**	KZNSUR	2010	sputum	Negative	SCI	mono	S	S	S	R	S	R	S	S	S	Beijing	LIN 2	Drug-resistant (other)	n/a
**TKK-01-0066**	KZNSUR	2010	sputum	Negative	SCI	susceptible	S	S	S	S	S	S	S	S	S	H37Rv	LIN 4	susceptible	n/a
**TKK-01-0067**	KZNSUR	2010	sputum	Negative	SCI	mono	S	S	S	S	S	S	S	R	S	Beijing	LIN 2	Drug-resistant (other)	n/a
**TKK-01-0068**	KZNSUR	2010	sputum	Negative	SCI	MDR	R	R	R	R	R	R	S	R	R	LAM4	LIN 4	MDR	n/a
**TKK-01-0069**	KZNSUR	2010	sputum	Positive	SCI	MDR	R	R	R	R	S	S	S	R	S	LAM4	LIN 4	MDR	n/a
**TKK-01-0070**	KZNSUR	2010	sputum	Negative	SCI	MDR	S	R	R	R	S	S	S	R	R	EAI1-SOM	LIN 1	Drug-resistant (other)	n/a
**TKK-01-0071**	KZNSUR	2010	sputum	Positive	SCI	MDR	S	R	S	R	S	R	S	R	S	LAM4	LIN 4	MDR	n/a
**TKK-01-0073**	KZNSUR	2010	sputum	Negative	SCI	MDR	S	R	R	R	S	R	S	R	R	LAM4	LIN 4	Drug-resistant (other)	n/a
**TKK-01-0074**	KZNSUR	2010	sputum	Positive	SCI	MDR	S	R	R	R	S	R	S	R	R	LAM4	LIN 4	MDR	n/a
**TKK-01-0075**	KZNSUR	2010	sputum	Positive	SCI	MDR	S	R	R	R	S	R	S	R	R	LAM4	LIN 4	MDR	n/a
**TKK-01-0076**	KZNSUR	2010	sputum	Positive	SCI	XDR	R	R	R	R	R	R	R	R	R	LAM4	LIN 4	XDR	Tugela Ferry XDR
**TKK-01-0077**	KZNSUR	2010	sputum	Negative	SCI	mono	S	S	S	S	S	S	S	S	R	LAM3	LIN 4	Drug-resistant (other)	n/a
**TKK-01-0078**	KZNSUR	2010	sputum	Positive	SCI	mono	S	S	S	S	S	S	S	R	S	Beijing	LIN 2	Drug-resistant (other)	n/a
**TKK-01-0079**	KZNSUR	2010	sputum	Positive	SCI	MDR	S	R	S	R	S	S	S	R	S	Beijing	LIN 2	susceptible	n/a
**TKK-01-0080**	KZNSUR	2010	sputum	Negative	SCI	poly	S	R	S	S	S	S	S	R	R	EAI1-SOM	LIN 1	Drug-resistant (other)	n/a
**TKK-01-0081**	KZNSUR	2008	sputum	Positive	SCI	susceptible	S	S	S	S	S	S	S	S	S	LAM4	LIN 4	susceptible	n/a
**TKK-01-0082**	KZNSUR	2008	sputum	Negative	SCI	susceptible	S	S	S	S	S	S	S	S	S	Beijing	LIN 2	susceptible	n/a
**TKK-01-0083**	KZNSUR	2008	sputum	Negative	SCI	susceptible	S	S	S	S	S	S	S	S	S	LAM3	LIN 4	susceptible	n/a
**TKK-01-0084**	KZNSUR	2008	sputum	Positive	SCI	susceptible	S	S	S	S	S	S	S	S	S	LAM4	LIN 4	susceptible	n/a
**TKK-01-0085**	KZNSUR	2008	sputum	Negative	SCI	susceptible	S	S	S	S	S	S	S	S	S	LAM4	LIN 4	susceptible	n/a

Specimen ID	Cohort	Year	Specimen Type	Smear Status	DNA isolation	Phenotypic DST Pattern	C	E	Et	H	K	P/N	O	R	S	Digital Spoligotype	Lineage	Genotypic DST pattern	Tugela Ferry vs. non-Tugela Ferry XDR
**TKK-01-0086**	KZNSUR	2008	sputum	Positive	SCI	susceptible	S	S	S	S	S	S	S	S	S	T1	LIN 4	susceptible	n/a
**TKK-01-0087**	KZNSUR	2008	sputum	Positive	SCI	susceptible	S	S	S	S	S	S	S	S	S	T2	LIN 4	susceptible	n/a
**TKK-01-0088**	KZNSUR	2008	sputum	Positive	SCI	susceptible	S	S	S	S	S	S	S	S	S	Beijing	LIN 2	susceptible	n/a
**TKK-01-0089**	KZNSUR	2008	sputum	Positive	SCI	susceptible	S	S	S	S	S	S	S	S	S	LAM3	LIN 4	susceptible	n/a
**TKK-01-0090**	KZNSUR	2008	sputum	Positive	SCI	susceptible	S	S	S	S	S	S	S	S	S	LAM3	LIN 4	susceptible	n/a
**TKK-01-0091**	KZNSUR	2008	sputum	Negative	SCI	susceptible	S	S	S	S	S	S	S	S	S	LAM4	LIN 4	susceptible	n/a
**TKK-01-0092**	KZNSUR	unknown	sputum	Negative	SCI	susceptible	S	S	S	S	S	S	S	S	S	Beijing	LIN 2	susceptible	n/a
**TKK-01-0093**	KZNSUR	2008	sputum	Positive	SCI	susceptible	S	S	S	S	S	S	S	S	S	Beijing	LIN 2	susceptible	n/a
**TKK-01-0094**	KZNSUR	2008	sputum	Positive	SCI	susceptible	S	S	S	S	S	S	S	S	S	Beijing	LIN 2	susceptible	n/a
**TKK_02_0001**	PROX	2010	sputum	Negative	SCI	XDR	R	R	R	R	R	R	R	R	S	Beijing	LIN 2	XDR	non-Tugela Ferry XDR
**TKK_02_0002**	PROX	2010	sputum	Negative	SCI	XDR	R	R	R	R	R	R	R	R	R	Beijing	LIN 2	XDR	non-Tugela Ferry XDR
**TKK_02_0004**	PROX	2010	sputum	Negative	SCI	XDR	R	R	R	R	R	R	R	R	R	LAM4	LIN 4	XDR	Tugela Ferry XDR
**TKK_02_0005**	PROX	2010	sputum	Negative	SCI	XDR	R	R	R	R	R	R	R	R	R	LAM4	LIN 4	XDR	Tugela Ferry XDR
**TKK_02_0006**	PROX	2010	sputum	Negative	SCI	XDR	R	R	R	R	R	R	R	R	R	LAM4	LIN 4	XDR	Tugela Ferry XDR
**TKK_02_0007**	PROX	2010	sputum	Negative	SCI	XDR	R	R	R	R	R	R	R	R	R	LAM4	LIN 4	XDR	Tugela Ferry XDR
**TKK_02_0008**	PROX	2010	sputum	Positive	SCI	XDR	R	R	R	R	R	R	R	R	R	LAM4	LIN 4	XDR	Tugela Ferry XDR
**TKK_02_0010**	PROX	2010	sputum	Positive	SCI	MDR	S	R	R	R	S	R	R	R	R	Beijing	LIN 2	MDR	n/a
**TKK_02_0012**	PROX	2010	sputum	Negative	SCI	XDR	R	R	R	R	R	R	R	R	R	LAM4	LIN 4	XDR	Tugela Ferry XDR
**TKK_02_0014**	PROX	2010	sputum	Positive	SCI	XDR	R	R	R	R	R	R	R	R	R	LAM4	LIN 4	XDR	Tugela Ferry XDR
**TKK_02_0016**	PROX	2010	sputum	Negative	SCI	XDR	R	R	R	R	R	R	R	R	R	LAM4	LIN 4	XDR	Tugela Ferry XDR
**TKK_02_0018**	PROX	2010	sputum	Negative	SCI	MDR	S	R	R	R	S	R	R	R	R	T3	LIN 4	MDR	n/a
**TKK_02_0020**	PROX	2010	sputum	Positive	SCI	MDR	S	S	S	R	R	S	S	R	R	LAM6	LIN 4	MDR	n/a
**TKK_02_0021**	PROX	2010	sputum	Negative	SCI	XDR	R	R	R	R	R	R	R	R	R	LAM4	LIN 4	XDR	Tugela Ferry XDR
**TKK_02_0023**	PROX	2010	sputum	Positive	SCI	MDR	S	R	R	R	S	R	S	R	R	LAM4	LIN 4	MDR	n/a
**TKK_02_0024**	PROX	2010	sputum	Negative	SCI	XDR	R	R	R	R	R	R	R	R	R	LAM4	LIN 4	XDR	Tugela Ferry XDR
**TKK_02_0025**	PROX	2010	sputum	Positive	SCI	MDR	S	R	R	R	S	R	R	R	R	S	LIN 4	MDR	n/a
**TKK_02_0027**	PROX	2010	sputum	Positive	SCI	XDR	S	R	R	R	R	R	R	R	R	X3	LIN 4	XDR	non-Tugela Ferry XDR

**Specimen ID**	**Cohort**	**Year**	**Specimen Type**	**Smear Status**	**DNA isolation**	**Phenotypic DST Pattern**	**C**	**E**	**Et**	**H**	**K**	**P/N**	**O**	**R**	**S**	**Digital Spoligotype**	**Lineage**	**Genotypic DST pattern**	**Tugela Ferry vs. non-Tugela Ferry XDR**
**TKK_02_0028**	PROX	2010	sputum	Negative	SCI	XDR	R	R	R	R	R	R	R	R	R	LAM4	LIN 4	XDR	Tugela Ferry XDR
**TKK_02_0030**	PROX	2010	sputum	Positive	SCI	XDR	R	R	R	R	R	R	R	R	R	LAM4	LIN 4	XDR	Tugela Ferry XDR
**TKK_02_0031**	PROX	2010	sputum	Negative	SCI	XDR	S	R	S	R	R	R	R	R	R	LAM4	LIN 4	XDR	Tugela Ferry XDR
**TKK_02_0035**	PROX	2010	sputum	Positive	SCI	XDR	S	R	R	R	R	R	R	R	R	X3	LIN 4	XDR	non-Tugela Ferry XDR
**TKK_02_0036**	PROX	2010	sputum	Negative	SCI	XDR	S	R	S	R	R	U	R	R	S	X3	LIN 4	XDR	non-Tugela Ferry XDR
**TKK_02_0037**	PROX	2010	sputum	Positive	SCI	XDR	R	R	R	R	S	R	R	R	R	X3	LIN 4	XDR	non-Tugela Ferry XDR
**TKK_02_0038**	PROX	2010	sputum	Positive	SCI	XDR	R	R	R	R	R	R	R	R	R	LAM4	LIN 4	XDR	Tugela Ferry XDR
**TKK_02_0040**	PROX	2011	sputum	Positive	SCI	XDR	R	R	R	R	R	R	R	R	R	LAM4	LIN 4	XDR	Tugela Ferry XDR
**TKK_02_0041**	PROX	2011	sputum	Positive	SCI	XDR	R	R	R	R	R	R	R	R	R	LAM4	LIN 4	XDR	Tugela Ferry XDR
**TKK_02_0042**	PROX	2011	sputum	Positive	SCI	MDR	S	R	R	R	S	S	R	R	R	S	LIN 4	MDR	n/a
**TKK_02_0043**	PROX	2011	sputum	Negative	SCI	XDR	R	R	R	R	R	R	R	R	R	LAM4	LIN 4	XDR	Tugela Ferry XDR
**TKK_02_0044**	PROX	2011	sputum	Negative	SCI	XDR	R	R	R	R	R	R	R	R	R	LAM4	LIN 4	XDR	Tugela Ferry XDR
**TKK_02_0046**	PROX	2011	sputum	Negative	SCI	poly	S	S	S	S	S	R	S	R	S	Beijing	LIN 2	Drug-resistant (other)	n/a
**TKK_02_0047**	PROX	2011	sputum	Positive	SCI	MDR	S	R	S	R	S	R	S	R	R	LAM4	LIN 4	MDR	n/a
**TKK_02_0048**	PROX	2011	sputum	Positive	SCI	XDR	R	R	R	R	R	R	R	R	R	LAM4	LIN 4	XDR	Tugela Ferry XDR
**TKK_02_0049**	PROX	2011	sputum	Positive	SCI	XDR	R	R	R	R	R	R	R	R	R	LAM4	LIN 4	XDR	Tugela Ferry XDR
**TKK_02_0050**	PROX	2011	sputum	Positive	SCI	XDR	R	R	R	R	R	R	R	R	R	LAM4	LIN 4	XDR	Tugela Ferry XDR
**TKK_02_0051**	PROX	2011	sputum	Positive	SCI	XDR	R	R	R	R	R	R	R	R	R	LAM4	LIN 4	XDR	Tugela Ferry XDR
**TKK_02_0052**	PROX	2011	sputum	Positive	SCI	XDR	R	R	R	R	R	R	R	R	R	LAM4	LIN 4	XDR	Tugela Ferry XDR
**TKK_02_0053**	PROX	2011	sputum	Negative	SCI	XDR	R	R	R	R	R	R	R	R	R	LAM4	LIN 4	XDR	Tugela Ferry XDR
**TKK_02_0055**	PROX	2011	sputum	Positive	SCI	XDR	R	R	R	R	R	R	R	R	R	Beijing	LIN 2	XDR	non-Tugela Ferry XDR
**TKK_02_0056**	PROX	2011	sputum	Positive	SCI	XDR	R	R	R	R	R	R	R	R	R	LAM4	LIN 4	XDR	Tugela Ferry XDR
**TKK_02_0058**	PROX	2011	sputum	Positive	SCI	XDR	R	R	R	R	R	R	R	R	R	LAM4	LIN 4	XDR	Tugela Ferry XDR
**TKK_02_0060**	PROX	2011	sputum	Negative	SCI	XDR	R	R	R	R	R	R	R	R	R	LAM4	LIN 4	XDR	Tugela Ferry XDR
**TKK_02_0062**	PROX	2011	sputum	Positive	SCI	MDR	S	R	R	R	S	R	R	R	R	CAS1-Kili	LIN 3	MDR	n/a
**TKK_02_0065**	PROX	2011	sputum	Positive	SCI	MDR	S	R	R	R	S	R	S	R	R	LAM4	LIN 4	MDR	n/a
**TKK_02_0066**	PROX	2011	sputum	Negative	SCI	MDR	S	R	S	R	S	S	S	R	R	S	LIN 4	MDR	n/a

**Specimen ID**	**Cohort**	**Year**	**Specimen Type**	**Smear Status**	**DNA isolation**	**Phenotypic DST Pattern**	**C**	**E**	**Et**	**H**	**K**	**P/N**	**O**	**R**	**S**	**Digital Spoligotype**	**Lineage**	**Genotypic DST pattern**	**Tugela Ferry vs. non-Tugela Ferry XDR**
**TKK_02_0067**	PROX	2011	sputum	Positive	SCI	MDR	S	R	R	R	R	S	S	R	S	S	LIN 4	MDR	n/a
**TKK_02_0068**	PROX	2011	sputum	Positive	SCI	MDR	S	R	R	R	R	S	S	R	S	T3	LIN 4	MDR	n/a
**TKK_02_0071**	PROX	2011	sputum	Negative	SCI	XDR	R	R	R	R	R	R	R	R	S	LAM4	LIN 4	XDR	Tugela Ferry XDR
**TKK_02_0073**	PROX	2011	sputum	Positive	SCI	MDR	S	R	R	R	S	R	R	R	S	LAM4	LIN 4	MDR	n/a
**TKK_02_0074**	PROX	2011	sputum	Negative	SCI	MDR	S	R	S	R	S	R	S	R	R	S	LIN 4	MDR	n/a
**TKK_02_0076**	PROX	2011	sputum	Positive	SCI	MDR	S	R	R	R	S	R	S	R	R	LAM4	LIN 4	MDR	n/a
**TKK_02_0079**	PROX	2012	sputum	Positive	SCI	MDR	S	R	R	R	S	S	R	R	R	LAM4	LIN 4	MDR	n/a
**TKK_02_0080**	PROX	2012	sputum	Positive	SCI	MDR	S	R	S	R	S	R	S	R	R	Beijing	LIN 2	MDR	n/a
**TKK_03_0015**	Phage	2012	sputum	Negative	SCI	susceptible	U	S	U	S	S	U	S	S	S	S	LIN 4	susceptible	n/a
**TKK_03_0020**	Phage	2012	sputum	Positive	SCI	susceptible	U	S	U	S	S	U	S	S	S	S	LIN 4	susceptible	n/a
**TKK_03_0021**	Phage	2013	sputum	Positive	SCI	susceptible	U	S	U	S	S	U	S	S	S	T1	LIN 4	susceptible	n/a
**TKK_03_0023**	Phage	2013	sputum	Negative	SCI	susceptible	U	S	U	S	S	U	S	S	S	LAM3	LIN 4	Drug-resistant (other)	n/a
**TKK_03_0024**	Phage	2013	sputum	Negative	SCI	poly	U	S	U	R	S	U	S	S	R	LAM3	LIN 4	Drug-resistant (other)	n/a
**TKK_03_0025**	Phage	2013	sputum	Positive	SCI	susceptible	U	S	U	S	S	U	S	S	S	Beijing	LIN 2	susceptible	n/a
**TKK_03_0026**	Phage	2013	sputum	Positive	SCI	susceptible	U	S	U	S	S	U	S	S	S	unknown	unknown	susceptible	n/a
**TKK_03_0027**	Phage	2013	sputum	Positive	SCI	susceptible	U	S	U	S	S	U	S	S	S	Beijing	LIN 2	susceptible	n/a
**TKK_03_0028**	Phage	2013	sputum	Positive	non-SCI	susceptible	U	S	U	S	S	U	S	S	S	Beijing	LIN 2	susceptible	n/a
**TKK_03_0029**	Phage	2013	sputum	Positive	SCI	susceptible	U	S	U	S	S	U	S	S	S	EAI1-SOM	LIN 1	susceptible	n/a
**TKK_03_0030**	Phage	2013	sputum	Positive	SCI	susceptible	U	S	U	S	S	U	S	S	S	T1	LIN 4	susceptible	n/a
**TKK_03_0031**	Phage	2013	sputum	Positive	SCI	susceptible	U	S	U	S	S	U	S	S	S	Beijing	LIN 2	Drug-resistant (other)	n/a
**TKK_03_0033**	Phage	2013	sputum	Positive	SCI	susceptible	U	S	U	S	S	U	S	S	S	CAS1-Delhi	LIN 3	susceptible	n/a
**TKK_03_0034**	Phage	2013	sputum	Positive	SCI	susceptible	U	S	U	S	S	U	S	S	S	Beijing	LIN 2	susceptible	n/a
**TKK_03_0035**	Phage	2013	sputum	Positive	non-SCI	susceptible	U	S	U	S	S	U	S	S	S	LAM3	LIN 4	susceptible	n/a
**TKK_03_0036**	Phage	2013	sputum	Positive	SCI	susceptible	U	S	U	S	S	U	S	S	S	LAM3	LIN 4	susceptible	n/a
**TKK_03_0037**	Phage	2013	sputum	Positive	non-SCI	susceptible	U	S	U	S	S	U	S	S	S	CAS1-Kili	LIN 3	susceptible	n/a
**TKK_03_0039**	Phage	2013	sputum	Positive	non-SCI	susceptible	U	S	U	S	S	U	S	S	S	Beijing	LIN 2	susceptible	n/a
**TKK_03_0040**	Phage	2013	sputum	Positive	non-SCI	susceptible	U	S	U	S	S	U	S	S	S	Beijing	LIN 2	susceptible	n/a

Specimen ID	Cohort	Year	Specimen Type	Smear Status	DNA isolation	Phenotypic DST Pattern	C	E	Et	H	K	P/N	O	R	S	Digital Spoligotype	Lineage	Genotypic DST pattern	Tugela Ferry vs. non-Tugela Ferry XDR
**TKK_03_0042**	Phage	2013	sputum	Positive	non-SCI	susceptible	U	S	U	S	S	U	S	S	S	T1	LIN 4	susceptible	n/a
**TKK_03_0043**	Phage	2013	sputum	Positive	SCI	susceptible	U	S	U	S	S	U	S	S	S	Beijing	LIN 2	susceptible	n/a
**TKK_03_0044**	Phage	2013	sputum	Positive	SCI	susceptible	U	S	U	S	S	U	S	S	S	T1	LIN 4	susceptible	n/a
**TKK_03_0045**	Phage	2013	sputum	Positive	SCI	susceptible	U	S	U	S	S	U	S	S	S	Beijing	LIN 2	susceptible	n/a
**TKK_03_0047**	Phage	2013	sputum	Negative	SCI	susceptible	U	S	U	S	S	U	S	S	S	Beijing	LIN 2	susceptible	n/a
**TKK_03_0050**	Phage	2013	sputum	Negative	SCI	susceptible	U	S	U	S	S	U	S	S	S	AMBIGOUS:T3 T2	LIN 4	susceptible	n/a
**TKK_03_0058**	Phage	2013	sputum	Positive	non-SCI	susceptible	U	S	U	S	S	U	S	S	S	Beijing	LIN 2	susceptible	n/a
**TKK_03_0059**	Phage	2013	sputum	Positive	non-SCI	susceptible	U	S	U	S	S	U	S	S	S	T1	LIN 4	susceptible	n/a
**TKK_03_0063**	Phage	2013	sputum	Positive	non-SCI	susceptible	U	S	U	S	S	U	S	S	S	X2	LIN 4	susceptible	n/a
**TKK_03_0064**	Phage	2013	sputum	Positive	SCI	susceptible	U	S	U	S	S	U	S	R	S	LAM3	LIN 4	Drug-resistant (other)	n/a
**TKK_03_0065**	Phage	2013	sputum	Positive	SCI	susceptible	U	S	U	S	S	U	S	S	S	Beijing	LIN 2	susceptible	n/a
**TKK_03_0072**	Phage	2013	sputum	Negative	SCI	susceptible	U	S	U	S	S	U	S	S	S	Beijing	LIN 2	susceptible	n/a
**TKK_03_0075**	Phage	2013	sputum	Positive	SCI	susceptible	U	S	U	S	S	U	S	S	S	Beijing	LIN 2	susceptible	n/a
**TKK_03_0078**	Phage	2013	sputum	Positive	SCI	susceptible	U	S	U	S	S	U	S	S	S	Beijing	LIN 2	susceptible	n/a
**TKK_03_0082**	Phage	2013	sputum	Negative	SCI	mono	U	S	U	R	S	U	S	S	S	LAM3	LIN 4	susceptible	n/a
**TKK_03_0083**	Phage	2013	sputum	Positive	SCI	susceptible	U	S	U	S	S	U	S	S	S	Beijing	LIN 2	susceptible	n/a
**TKK_03_0089**	Phage	2013	sputum	Positive	SCI	susceptible	U	S	U	S	S	U	S	S	S	LAM4	LIN 4	susceptible	n/a
**TKK_03_0090**	Phage	2013	sputum	Negative	non-SCI	susceptible	U	S	U	S	S	U	S	S	S	Beijing	LIN 2	susceptible	n/a
**TKK_03_0092**	Phage	2013	sputum	Negative	SCI	susceptible	U	S	U	S	S	U	S	S	S	LAM3	LIN 4	susceptible	n/a
**TKK_03_0094**	Phage	2013	sputum	Positive	non-SCI	susceptible	U	S	U	S	S	U	S	S	S	S	LIN 4	susceptible	n/a
**TKK_03_0098**	Phage	2013	sputum	Negative	non-SCI	susceptible	U	S	U	S	S	U	S	S	S	S	LIN 4	susceptible	n/a
**TKK_03_0099**	Phage	2013	sputum	Positive	non-SCI	susceptible	U	S	U	S	S	U	S	S	S	X1	LIN 4	susceptible	n/a
**TKK_03_0100**	Phage	2013	sputum	Positive	non-SCI	susceptible	U	S	U	S	S	U	S	S	S	Beijing	LIN 2	susceptible	n/a
**TKK_03_0101**	Phage	2013	sputum	Positive	non-SCI	mono	U	S	U	R	S	U	S	S	S	H1	LIN 4	Drug-resistant (other)	n/a
**TKK_03_0102**	Phage	2013	sputum	Positive	non-SCI	susceptible	U	S	U	S	S	U	S	S	S	LAM3	LIN 4	susceptible	n/a
**TKK_03_0103**	Phage	2013	sputum	Negative	SCI	susceptible	U	S	U	S	S	U	S	S	S	H1	LIN 4	susceptible	n/a
**TKK_03_0105**	Phage	2013	sputum	Negative	SCI	susceptible	U	S	U	S	S	U	S	S	S	LAM1,LAM3	LIN 4	susceptible	n/a

Specimen ID	Cohort	Year	Specimen Type	Smear Status	DNA isolation	Phenotypic DST Pattern	C	E	Et	H	K	P/N	O	R	S	Digital Spoligotype	Lineage	Genotypic DST pattern	Tugela Ferry vs. non-Tugela Ferry XDR
**TKK_03_0108**	Phage	2013	sputum	Positive	non-SCI	susceptible	U	S	U	S	S	U	S	S	S	X3	LIN 4	susceptible	n/a
**TKK_03_0109**	Phage	2013	sputum	Negative	non-SCI	susceptible	U	S	U	S	S	U	S	S	S	Beijing	LIN 2	susceptible	n/a
**TKK_03_0111**	Phage	2013	sputum	Positive	non-SCI	susceptible	U	S	U	S	S	U	S	S	S	Beijing	LIN 2	susceptible	n/a
**TKK_03_0112**	Phage	2013	sputum	Positive	non-SCI	susceptible	U	S	U	S	S	U	S	S	S	Beijing	LIN 2	susceptible	n/a
**TKK_03_0114**	Phage	2013	sputum	Positive	SCI	susceptible	U	S	U	S	S	U	S	S	S	Beijing	LIN 2	susceptible	n/a
**TKK_03_0115**	Phage	2013	sputum	Positive	non-SCI	susceptible	U	S	U	S	S	U	S	S	S	LAM6	LIN 4	susceptible	n/a
**TKK_03_0118**	Phage	2013	sputum	Positive	non-SCI	susceptible	U	S	U	S	S	U	S	S	S	Beijing	LIN 2	susceptible	n/a
**TKK_03_0149**	Phage	2013	sputum	Positive	non-SCI	susceptible	U	S	U	S	S	U	S	S	S	Beijing	LIN 2	susceptible	n/a
**TKK_03_0150**	Phage	2013	sputum	Positive	non-SCI	susceptible	U	S	U	S	S	U	S	S	S	X3	LIN 4	susceptible	n/a
**TKK_03_0153**	Phage	2013	sputum	Positive	non-SCI	susceptible	U	S	U	S	S	U	S	S	S	X3	LIN 4	susceptible	n/a
**TKK_03_0154**	Phage	2013	sputum	Positive	non-SCI	susceptible	U	S	U	S	S	U	S	S	S	Beijing	LIN 2	susceptible	n/a
**TKK_03_0156**	Phage	2013	sputum	Positive	non-SCI	susceptible	U	S	U	S	S	U	S	S	S	Beijing	LIN 2	susceptible	n/a
**TKK_03_0158**	Phage	2013	sputum	Positive	non-SCI	susceptible	U	S	U	S	S	U	S	S	S	AMBIGOUS:T2 X1	LIN 4	susceptible	n/a
**TKK_03_0159**	Phage	2013	sputum	Positive	non-SCI	susceptible	U	S	U	S	S	U	S	S	S	LAM4	LIN 4	susceptible	n/a
**TKK_03_0160**	Phage	2013	sputum	Positive	non-SCI	susceptible	U	S	U	S	S	U	S	S	S	Beijing	LIN 2	susceptible	n/a
**TKK_04_0001**	NHLS	2013	sputum	unknown	SCI	poly	U	U	U	R	S	U	S	S	R	Beijing	LIN 2	MDR	n/a
**TKK_04_0002**	NHLS	2013	sputum	unknown	SCI	poly	U	U	U	R	S	U	S	S	R	T1	LIN 4	MDR	n/a
**TKK_04_0003**	NHLS	2013	sputum	unknown	SCI	mono	U	U	U	R	S	U	S	S	S	EAI1-SOM	LIN 1	Drug-resistant (other)	n/a
**TKK_04_0005**	NHLS	2013	sputum	Positive	SCI	poly	U	U	U	R	S	U	R	S	R	EAI1-SOM	LIN 1	MDR	n/a
**TKK_04_0006**	NHLS	2013	sputum	unknown	SCI	MDR	U	U	U	R	S	U	S	R	R	Beijing	LIN 2	Drug-resistant (other)	n/a
**TKK_04_0007**	NHLS	2013	sputum	Negative	SCI	MDR	U	U	U	R	S	U	R	R	S	S	LIN 4	MDR	n/a
**TKK_04_0008**	NHLS	2013	sputum	Positive	SCI	MDR	U	U	U	R	S	U	R	R	R	S	LIN 4	MDR	n/a
**TKK_04_0013**	NHLS	2013	sputum	unknown	SCI	MDR	U	U	U	R	S	U	R	R	R	LAM4	LIN 4	Drug-resistant (other)	n/a
**TKK_04_0014**	NHLS	2013	sputum	Negative	SCI	mono	U	U	U	S	S	U	S	R	S	Beijing	LIN 2	Drug-resistant (other)	n/a
**TKK_04_0015**	NHLS	2013	sputum	Positive	SCI	mono	U	U	U	S	S	U	S	R	S	LAM3	LIN 4	Drug-resistant (other)	n/a
**TKK_04_0017**	NHLS	2013	sputum	unknown	SCI	poly	U	U	U	R	S	U	S	S	R	Beijing	LIN 2	MDR	n/a
**TKK_04_0018**	NHLS	2013	sputum	Positive	SCI	MDR	U	U	U	R	S	U	S	R	R	Beijing	LIN 2	susceptible	n/a

**Specimen ID**	**Cohort**	**Year**	**Specimen Type**	**Smear Status**	**DNA isolation**	**Phenotypic DST Pattern**	**C**	**E**	**Et**	**H**	**K**	**P/N**	**O**	**R**	**S**	**Digital Spoligotype**	**Lineage**	**Genotypic DST pattern**	**Tugela Ferry vs. non-Tugela Ferry XDR**
**TKK_04_0019**	NHLS	2013	sputum	unknown	SCI	MDR	U	U	U	R	S	U	S	R	R	Beijing	LIN 2	MDR	n/a
**TKK_04_0020**	NHLS	2013	sputum	Positive	SCI	MDR	U	U	U	R	S	U	R	R	R	EAI1-SOM	LIN 1	MDR	n/a
**TKK_04_0021**	NHLS	2013	pleura	Positive	SCI	MDR	U	U	U	R	S	U	S	R	R	S	LIN 4	MDR	n/a
**TKK_04_0022**	NHLS	2013	sputum	unknown	SCI	MDR	U	U	U	R	S	U	S	R	R	X3	LIN 4	MDR	n/a
**TKK_04_0023**	NHLS	2013	sputum	unknown	SCI	MDR	U	U	U	R	S	U	S	R	R	Beijing	LIN 2	MDR	n/a
**TKK_04_0024**	NHLS	2013	sputum	Positive	SCI	MDR	U	U	U	R	S	U	S	R	R	S	LIN 4	MDR	n/a
**TKK_04_0029**	NHLS	2013	pleural fluid	unknown	SCI	susceptible	U	U	U	S	S	U	S	S	S	LAM3	LIN 4	susceptible	n/a
**TKK_04_0030**	NHLS	2013	sputum	unknown	SCI	susceptible	U	U	U	S	S	U	S	S	S	S,T1	LIN 4	susceptible	n/a
**TKK_04_0031**	NHLS	2013	blood	unknown	SCI	susceptible	U	U	U	S	S	U	S	S	S	T4,T1	LIN 4	susceptible	n/a
**TKK_04_0033**	NHLS	2013	blood	unknown	SCI	susceptible	U	U	U	S	S	U	S	S	S	Beijing	LIN 2	susceptible	n/a
**TKK_04_0034**	NHLS	2013	sputum	unknown	SCI	MDR	U	U	U	R	S	U	R	R	R	X3	LIN 4	MDR	n/a
**TKK_04_0036**	NHLS	2013	sputum	unknown	SCI	MDR	U	U	U	R	S	U	R	R	R	S	LIN 4	MDR	n/a
**TKK_04_0037**	NHLS	2013	sputum	unknown	SCI	MDR	U	U	U	R	S	U	R	R	R	S	LIN 4	MDR	n/a
**TKK_04_0038**	NHLS	2013	sputum	Positive	SCI	MDR	U	U	U	R	S	U	R	R	R	LAM4	LIN 4	MDR	n/a
**TKK_04_0039**	NHLS	2013	sputum	unknown	SCI	MDR	U	U	U	R	S	U	R	R	R	LAM4	LIN 4	MDR	n/a
**TKK_04_0040**	NHLS	2013	sputum	unknown	SCI	poly	U	U	U	R	S	U	R	S	R	X2	LIN 4	MDR	n/a
**TKK_04_0042**	NHLS	2013	sputum	unknown	SCI	MDR	U	U	U	R	S	U	R	R	R	S	LIN 4	MDR	n/a
**TKK_04_0043**	NHLS	2013	Sputum	Positive	SCI	MDR	U	U	U	R	R	U	S	R	S	LAM4	LIN 4	MDR	n/a
**TKK_04_0044**	NHLS	2013	sputum	unknown	SCI	MDR	U	U	U	R	S	U	R	R	R	LAM3	LIN 4	MDR	n/a
**TKK_04_0045**	NHLS	2013	sputum	unknown	SCI	MDR	U	U	U	R	S	U	R	R	R	S	LIN 4	MDR	n/a
**TKK_04_0046**	NHLS	2013	sputum	unknown	SCI	MDR	U	U	U	R	S	U	R	R	R	S	LIN 4	MDR	n/a
**TKK_04_0047**	NHLS	2013	sputum	unknown	SCI	poly	U	U	U	R	R	U	S	S	S	LAM3	LIN 4	MDR	n/a
**TKK_04_0048**	NHLS	2013	sputum	unknown	SCI	MDR	U	U	U	R	R	U	S	R	R	LAM4	LIN 4	MDR	n/a
**TKK_04_0051**	NHLS	2013	sputum	unknown	SCI	MDR	U	U	U	R	S	U	R	R	R	LAM4	LIN 4	MDR	n/a
**TKK_04_0054**	NHLS	2013	sputum	unknown	SCI	MDR	U	U	U	R	S	U	R	R	R	Beijing	LIN 2	MDR	n/a
**TKK_04_0059**	NHLS	2013	sputum	Positive	SCI	MDR	U	U	U	R	S	U	R	R	R	LAM4	LIN 4	MDR	n/a
**TKK_04_0060**	NHLS	2013	sputum	unknown	SCI	MDR	U	U	U	R	S	U	R	R	S	LAM4	LIN 4	MDR	n/a

Specimen ID	Cohort	Year	Specimen Type	Smear Status	DNA isolation	Phenotypic DST Pattern	C	E	Et	H	K	P/N	O	R	S	Digital Spoligotype	Lineage	Genotypic DST pattern	Tugela Ferry vs. non-Tugela Ferry XDR
**TKK_04_0061**	NHLS	2013	sputum	unknown	SCI	MDR	U	U	U	R	S	U	R	R	R	T1	LIN 4	MDR	n/a
**TKK_04_0062**	NHLS	2013	sputum	unknown	SCI	MDR	U	U	U	R	S	U	R	R	S	LAM4	LIN 4	MDR	n/a
**TKK_04_0064**	NHLS	2013	sputum	unknown	SCI	MDR	U	U	U	R	S	U	R	R	S	LAM4	LIN 4	Drug-resistant (other)	n/a
**TKK_04_0066**	NHLS	2013	sputum	unknown	SCI	MDR	U	U	U	R	S	U	R	R	R	LAM4	LIN 4	Drug-resistant (other)	n/a
**TKK_04_0067**	NHLS	2013	sputum	Positive	SCI	MDR	U	U	U	R	S	U	R	R	S	S	LIN 4	MDR	n/a
**TKK_04_0068**	NHLS	2013	sputum	unknown	SCI	MDR	U	U	U	R	S	U	R	R	R	AMBIGOUS:T2 X1	LIN 4	MDR	n/a
**TKK_04_0069**	NHLS	2013	sputum	Positive	SCI	MDR	U	U	U	R	S	U	R	R	R	X2	LIN 4	MDR	n/a
**TKK_04_0070**	NHLS	2013	pus	unknown	SCI	MDR	U	U	U	R	S	U	R	R	S	LAM4	LIN 4	MDR	n/a
**TKK_04_0071**	NHLS	2013	sputum	Negative	SCI	MDR	U	U	U	R	S	U	R	R	R	X3	LIN 4	MDR	n/a
**TKK_04_0074**	NHLS	2013	sputum	unknown	SCI	MDR	U	U	U	R	S	U	R	R	R	S	LIN 4	MDR	n/a
**TKK_04_0075**	NHLS	2013	sputum	unknown	SCI	MDR	U	U	U	R	S	U	R	R	R	T1	LIN 4	MDR	n/a
**TKK_04_0078**	NHLS	2013	sputum	unknown	SCI	MDR	U	U	U	R	S	U	R	R	S	LAM4	LIN 4	Drug-resistant (other)	n/a
**TKK_04_0080**	NHLS	2013	sputum	Positive	SCI	MDR	U	U	U	R	S	U	S	R	R	CAS	LIN 3	Drug-resistant (other)	n/a
**TKK_04_0081**	NHLS	2013	sputum	unknown	SCI	MDR	U	U	U	R	S	U	S	R	R	LAM4	LIN 4	MDR	n/a
**TKK_04_0082**	NHLS	2013	sputum	unknown	SCI	mono	U	U	U	R	S	U	S	S	S	LAM4	LIN 4	MDR	n/a
**TKK_04_0083**	NHLS	2013	sputum	unknown	SCI	MDR	U	U	U	R	S	U	R	R	R	LAM4	LIN 4	Drug-resistant (other)	n/a
**TKK_04_0084**	NHLS	2013	sputum	unknown	SCI	MDR	U	U	U	R	S	U	S	R	R	Beijing	LIN 2	Drug-resistant (other)	n/a
**TKK_04_0085**	NHLS	2013	sputum	Positive	SCI	MDR	U	U	U	R	S	U	S	R	R	EAI1-SOM	LIN 1	Drug-resistant (other)	n/a
**TKK_04_0086**	NHLS	2013	sputum	Positive	SCI	mono	U	U	U	R	S	U	S	S	S	Beijing	LIN 2	MDR	n/a
**TKK_04_0089**	NHLS	2013	sputum	unknown	SCI	mono	U	U	U	R	R	U	S	S	S	Beijing	LIN 2	Drug-resistant (other)	n/a
**TKK_04_0090**	NHLS	2013	sputum	unknown	SCI	mono	U	U	U	S	S	U	S	R	S	LAM4	LIN 4	MDR	n/a
**TKK_04_0094**	NHLS	2013	sputum	unknown	SCI	MDR	U	U	U	R	S	U	S	R	S	LAM3	LIN 4	Drug-resistant (other)	n/a
**TKK_04_0095**	NHLS	2013	sputum	unknown	SCI	MDR	U	U	U	R	S	U	S	R	R	LAM4	LIN 4	MDR	n/a
**TKK_04_0096**	NHLS	2013	sputum	unknown	SCI	MDR	U	U	U	R	S	U	S	R	R	LAM4	LIN 4	MDR	n/a
**TKK_04_0097**	NHLS	2013	sputum	Negative	SCI	MDR	U	U	U	R	S	U	S	R	S	LAM4	LIN 4	MDR	n/a
**TKK_04_0098**	NHLS	2013	sputum	unknown	SCI	MDR	U	U	U	R	S	U	S	R	S	Beijing	LIN 2	Drug-resistant (other)	n/a
**TKK_04_0099**	NHLS	2013	sputum	unknown	SCI	MDR	U	U	U	R	S	U	S	R	S	LAM4	LIN 4	Drug-resistant (other)	n/a

Specimen ID	Cohort	Year	Specimen Type	Smear Status	DNA isolation	Phenotypic DST Pattern	C	E	Et	H	K	P/N	O	R	S	Digital Spoligotype	Lineage	Genotypic DST pattern	Tugela Ferry vs. non-Tugela Ferry XDR
**TKK_04_0103**	NHLS	2013	sputum	unknown	SCI	XDR	U	U	U	R	R	U	R	R	S	X3	LIN 4	XDR	non-Tugela Ferry XDR
**TKK_04_0104**	NHLS	2013	sputum	unknown	SCI	XDR	U	U	U	R	R	U	R	R	R	LAM4	LIN 4	XDR	Tugela Ferry XDR
**TKK_04_0105**	NHLS	2013	sputum	Negative	SCI	XDR	U	U	U	R	R	U	R	R	R	LAM4	LIN 4	XDR	Tugela Ferry XDR
**TKK_04_0106**	NHLS	2013	sputum	Positive	SCI	XDR	U	U	U	R	R	U	R	R	R	LAM4	LIN 4	XDR	Tugela Ferry XDR
**TKK_04_0107**	NHLS	2013	sputum	Negative	SCI	XDR	U	U	U	R	R	U	R	R	R	Beijing	LIN 2	XDR	non-Tugela Ferry XDR
**TKK_04_0108**	NHLS	2013	sputum	unknown	SCI	XDR	U	U	U	R	R	U	R	R	R	LAM4	LIN 4	XDR	Tugela Ferry XDR
**TKK_04_0109**	NHLS	2013	sputum	unknown	SCI	XDR	U	U	U	R	R	U	R	R	R	LAM4	LIN 4	XDR	Tugela Ferry XDR
**TKK_04_0112**	NHLS	2013	sputum	Negative	SCI	XDR	U	U	U	R	R	U	R	R	R	LAM4	LIN 4	XDR	Tugela Ferry XDR
**TKK_04_0113**	NHLS	2013	sputum	unknown	SCI	XDR	U	U	U	R	R	U	R	R	S	X3	LIN 4	XDR	non-Tugela Ferry XDR
**TKK_04_0114**	NHLS	2013	sputum	Positive	SCI	XDR	U	U	U	R	R	U	R	R	R	Beijing	LIN 2	XDR	non-Tugela Ferry XDR
**TKK_04_0117**	NHLS	2013	sputum	unknown	SCI	XDR	U	U	U	R	R	U	R	R	R	AMBIGOUS:LAM5 LAM4	LIN 4	XDR	non-Tugela Ferry XDR
**TKK_04_0118**	NHLS	2013	sputum	unknown	SCI	XDR	U	U	U	R	R	U	R	R	R	LAM4	LIN 4	XDR	Tugela Ferry XDR
**TKK_04_0120**	NHLS	2013	sputum	unknown	SCI	XDR	U	U	U	R	R	U	R	R	R	EAI1-SOM	LIN 1	XDR	non-Tugela Ferry XDR
**TKK_04_0122**	NHLS	2013	sputum	unknown	SCI	XDR	U	U	U	R	R	U	R	R	R	LAM4	LIN 4	XDR	Tugela Ferry XDR
**TKK_04_0123**	NHLS	2013	sputum	unknown	SCI	XDR	U	U	U	R	R	U	R	R	S	LAM4	LIN 4	XDR	Tugela Ferry XDR
**TKK_04_0124**	NHLS	2013	sputum	Positive	SCI	XDR	U	U	U	R	R	U	R	R	R	LAM4	LIN 4	XDR	Tugela Ferry XDR
**TKK_04_0125**	NHLS	2013	sputum	Negative	SCI	MDR	U	U	U	R	R	U	S	R	R	T1	LIN 4	MDR	n/a
**TKK_04_0126**	NHLS	2013	Sputum	unknown	SCI	MDR	U	U	U	R	R	U	S	R	R	Beijing	LIN 2	MDR	n/a
**TKK_04_0129**	NHLS	2013	sputum	Negative	SCI	XDR	U	U	U	R	R	U	R	R	R	LAM4	LIN 4	XDR	Tugela Ferry XDR
**TKK_04_0130**	NHLS	2013	sputum	Positive	SCI	MDR	U	U	U	R	S	U	S	R	R	LAM4	LIN 4	MDR	n/a
**TKK_04_0131**	NHLS	2013	sputum	unknown	SCI	MDR	U	U	U	R	R	U	S	R	R	Beijing	LIN 2	MDR	n/a
**TKK_04_0132**	NHLS	2013	sputum	unknown	SCI	XDR	U	U	U	R	R	U	R	R	R	LAM4	LIN 4	XDR	Tugela Ferry XDR
**TKK_04_0134**	NHLS	2013	sputum	unknown	SCI	poly	U	U	U	R	S	U	S	S	R	Beijing	LIN 2	MDR	n/a
**TKK_04_0136**	NHLS	2013	sputum	Positive	SCI	poly	U	U	U	R	S	U	S	S	R	Beijing	LIN 2	MDR	n/a
**TKK_04_0137**	NHLS	2013	sputum	unknown	SCI	XDR	U	U	U	R	R	U	R	R	R	LAM4	LIN 4	XDR	Tugela Ferry XDR
**TKK_04_0139**	NHLS	2013	sputum	unknown	SCI	MDR	U	U	U	R	S	U	S	R	R	CAS1-Kili	LIN 3	MDR	n/a
**TKK_04_0140**	NHLS	2013	sputum	unknown	SCI	MDR	U	U	U	R	R	U	S	R	R	LAM3	LIN 4	Drug-resistant (other)	n/a

Specimen ID	Cohort	Year	Specimen Type	Smear Status	DNA isolation	Phenotypic DST Pattern	C	E	Et	H	K	P/N	O	R	S	Digital Spoligotype	Lineage	Genotypic DST pattern	Tugela Ferry vs. non-Tugela Ferry XDR
**TKK_04_0141**	NHLS	2013	sputum	Negative	SCI	MDR	U	U	U	R	S	U	S	R	S	LAM4	LIN 4	MDR	n/a
**TKK_04_0145**	NHLS	2013	sputum	unknown	SCI	MDR	U	U	U	R	S	U	S	R	R	LAM4	LIN 4	MDR	n/a
**TKK_04_0148**	NHLS	2013	sputum	unknown	SCI	susceptible	U	U	U	S	S	U	S	S	S	CAS1-Delhi	LIN 3	Drug-resistant (other)	n/a
**TKK_04_0149**	NHLS	2013	sputum	unknown	SCI	susceptible	U	U	U	S	S	U	S	S	S	Beijing	LIN 2	Drug-resistant (other)	n/a
**TKK_04_0150**	NHLS	2013	sputum	Negative	SCI	MDR	U	U	U	R	S	U	S	R	S	LAM4	LIN 4	MDR	n/a
**TKK_04_0153**	NHLS	2013	sputum	Negative	SCI	poly	U	U	U	R	S	U	S	S	R	LAM9	LIN 4	MDR	n/a
**TKK_04_0155**	NHLS	2013	sputum	unknown	SCI	MDR	U	U	U	R	S	U	S	R	S	T1	LIN 4	Drug-resistant (other)	n/a
**TKK_04_0157**	NHLS	2013	sputum	Positive	SCI	MDR	U	U	U	R	S	U	S	R	S	LAM4	LIN 4	MDR	n/a
**TKK_04_0158**	NHLS	2013	CSF	unknown	SCI	MDR	U	U	U	R	S	U	S	R	R	LAM4	LIN 4	Drug-resistant (other)	n/a
**TKK_04_0159**	NHLS	2013	sputum	unknown	SCI	mono	U	U	U	R	S	U	S	S	S	Beijing	LIN 2	MDR	n/a
**TKK_05MA_0004**	CUBS	2013	sputum	Positive	non-SCI	MDR	U	R	U	R	S	U	S	R	R	S	LIN 4	MDR	n/a
**TKK_05MA_0009**	CUBS	2013	sputum	Positive	non-SCI	MDR	U	S	U	R	S	U	S	R	S	S	LIN 4	MDR	n/a
**TKK_05MA_0033**	CUBS	2013	sputum	Positive	non-SCI	MDR	U	R	U	R	S	U	S	R	R	S	LIN 4	MDR	n/a
**TKK_05MA_0035**	CUBS	2013	sputum	Positive	non-SCI	MDR	U	R	U	R	S	U	S	R	R	S	LIN 4	MDR	n/a
**TKK_05MA_0037**	CUBS	2013	sputum	Positive	non-SCI	MDR	U	R	U	R	S	U	S	R	S	LAM9	LIN 4	MDR	n/a
**TKK_05MA_0040**	CUBS	2013	sputum	Negative	non-SCI	MDR	U	R	U	R	S	U	S	R	S	S	LIN 4	MDR	n/a
**TKK_05MA_0051**	CUBS	2013	sputum	Positive	non-SCI	MDR	U	R	U	R	S	U	S	R	S	LAM4	LIN 4	MDR	n/a
**TKK_05MA_2005**	CUBS	2013	sputum	Positive	non-SCI	XDR	U	R	U	R	R	U	R	R	R	LAM4	LIN 4	XDR	Tugela Ferry XDR
**TKK_05MA_2008**	CUBS	2013	sputum	Positive	non-SCI	XDR	U	R	U	R	R	U	R	R	R	LAM4	LIN 4	XDR	Tugela Ferry XDR
**TKK_05MA_2015**	CUBS	2013	sputum	Negative	non-SCI	XDR	U	S	U	R	R	U	R	R	R	T1	LIN 4	XDR	non-Tugela Ferry XDR
**TKK_05SA_0010**	CUBS	2013	sputum	Negative	SCI	MDR	U	S	U	R	S	U	S	R	R	LAM3	LIN 4	MDR	n/a
**TKK_05SA_0011**	CUBS	2013	sputum	Positive	SCI	MDR	U	U	U	R	S	U	S	R	S	LAM4	LIN 4	MDR	n/a
**TKK_05SA_0014**	CUBS	2013	sputum	Positive	SCI	MDR	U	S	U	R	S	U	S	R	S	LAM4	LIN 4	MDR	n/a
**TKK_05SA_0016**	CUBS	2013	sputum	Positive	SCI	XDR	U	R	U	R	R	U	R	R	R	S	LIN 4	XDR	non-Tugela Ferry XDR
**TKK_05SA_0018**	CUBS	2013	sputum	Positive	SCI	XDR	U	R	U	R	R	U	R	R	R	X3	LIN 4	XDR	non-Tugela Ferry XDR
**TKK_05SA_0019**	CUBS	2013	sputum	Positive	SCI	mono	U	S	U	S	S	U	S	R	S	Beijing	LIN 2	Drug-resistant (other)	n/a
**TKK_05SA_0020**	CUBS	2013	sputum	Positive	SCI	MDR	U	R	U	R	S	U	S	R	S	T1	LIN 4	MDR	n/a

Specimen ID	Cohort	Year	Specimen Type	Smear Status	DNA isolation	Phenotypic DST Pattern	C	E	Et	H	K	P/N	O	R	S	Digital Spoligotype	Lineage	Genotypic DST pattern	Tugela Ferry vs. non-Tugela Ferry XDR
**TKK_05SA_0021**	CUBS	2013	sputum	Positive	SCI	susceptible	U	S	U	S	S	U	S	S	S	X3	LIN 4	Drug-resistant (other)	n/a
**TKK_05SA_0024**	CUBS	2013	sputum	Positive	SCI	susceptible	U	S	U	S	S	U	S	S	S	LAM3	LIN 4	Drug-resistant (other)	n/a
**TKK_05SA_0025**	CUBS	2013	sputum	Positive	SCI	MDR	U	S	U	R	S	U	S	R	R	LAM3	LIN 4	MDR	n/a
**TKK_05SA_0041**	CUBS	2013	sputum	Positive	SCI	MDR	U	R	U	R	S	U	S	R	R	T3,T1	LIN 4	MDR	n/a
**TKK_05SA_0042**	CUBS	2013	sputum	Positive	SCI	MDR	U	S	U	R	S	U	R	R	S	LAM4	LIN 4	MDR	n/a
**TKK_05SA_0043**	CUBS	2013	sputum	Positive	SCI	XDR	U	R	U	R	R	U	R	R	R	LAM4	LIN 4	XDR	Tugela Ferry XDR
**TKK_05SA_0044**	CUBS	2013	sputum	Positive	SCI	MDR	U	S	U	R	S	U	S	R	R	T3	LIN 4	MDR	n/a
**TKK_05SA_0046**	CUBS	2013	sputum	Positive	SCI	mono	U	S	U	S	S	U	S	R	S	Beijing	LIN 2	Drug-resistant (other)	n/a
**TKK_05SA_0048**	CUBS	2013	sputum	Positive	SCI	MDR	U	S	U	R	S	U	S	R	S	LAM4	LIN 4	MDR	n/a
**TKK_05SA_0050**	CUBS	2013	sputum	Positive	SCI	MDR	U	S	U	R	S	U	S	R	S	CAS1-Kili	LIN 3	MDR	n/a
**TKK_05SA_0052**	CUBS	2013	sputum	Positive	SCI	MDR	U	S	U	R	S	U	S	R	S	Beijing	LIN 2	MDR	n/a
**TKK_05SA_0054**	CUBS	2013	sputum	Positive	SCI	MDR	U	R	U	R	S	U	S	R	R	T1	LIN 4	MDR	n/a
**TKK_05SA_0055**	CUBS	2013	sputum	Positive	SCI	MDR	U	S	U	R	S	U	S	R	R	LAM4	LIN 4	MDR	n/a
