# Supplementary material for: Evolution of Extensively Drug-Resistant Tuberculosis over Four Decades: Whole Genome Sequencing and Dating Analysis of Mycobacterium tuberculosis Isolates from KwaZulu-Natal
Source: PLoS Med. 2015 Sep 29;12(9):e1001880. doi: 10.1371/journal.pmed.1001880 (PMC4587932; doi:10.1371/journal.pmed.1001880)
Supplement: S2 Table — A linkage analysis identified 11 drug-resistant clones in the entire dataset. The largest clone contained 50 members of the LAM4 spoligotype; this spoligotype was subsequently identified as the Tugela Ferry XDR Clone. Within the LAM4 spoligotype, there were three additional clones identified, and clones were also identified in five other spoligotypes. All clone members were noted to be drug resistant, indicating recent person-to-person transmission of drug-resistant TB. See S1 Methods for definition of a clone. (PDF) [file pmed.1001880.s007.pdf]

Clone Name	Spoligotype	# of Isolates	Phenotypic DST	Figure S2 Identifier
Tugela Ferry Clone	LAM4	50	XDR	10-AAI
LAM4-2	LAM4	4	MDR/XDR	10-ABK
LAM4-3	LAM4	5	MDR	10-ADI
LAM4-4	LAM4	11	mono/poly/MDR	10-ABQ
S-1	S	13	MDR/XDR	10-ABR
S-2	S	5	MDR	10-ABZ
S-3	S	3	MDR	10-ABJ
Beijing	Beijing	5	MDR/XDR	10-AAG
X3	X3	5	MDR/XDR	10-ABN
T3	T3	3	MDR	10-AFO
EAI1-SOM	EAI1-SOM	3	poly/MDR	10-AGR
