# Supplementary material for: Evolution of Extensively Drug-Resistant Tuberculosis over Four Decades: Whole Genome Sequencing and Dating Analysis of Mycobacterium tuberculosis Isolates from KwaZulu-Natal
Source: PLoS Med. 2015 Sep 29;12(9):e1001880. doi: 10.1371/journal.pmed.1001880 (PMC4587932; doi:10.1371/journal.pmed.1001880)
Supplement: S3 Table — Drug-resistant strains belonged to many distinct spoligotypes, which highlights the diversity of the drug resistance epidemic in this region. With a parsimony-based analysis, we quantified the independent evolutionary gains of genotypic MDR and XDR in our 340-strain dataset. (PDF) [file pmed.1001880.s008.pdf]

DST pattern	# of Strains	# of predicted spoligotypes	# of independent evolutionary gains
Susceptible	89	15	n/a
Mono drug-resistant	23	8	Not calculated
Poly drug-resistant	19	9	Not calculated
MDR *sensu stricto*	141	12	56
XDR	68	7	9
