# Supplementary material for: Evolution of Extensively Drug-Resistant Tuberculosis over Four Decades: Whole Genome Sequencing and Dating Analysis of Mycobacterium tuberculosis Isolates from KwaZulu-Natal
Source: PLoS Med. 2015 Sep 29;12(9):e1001880. doi: 10.1371/journal.pmed.1001880 (PMC4587932; doi:10.1371/journal.pmed.1001880)
Supplement: S6 Table — ahpC and ubiA mutations were ordered with respect to genotypic isoniazid and ethambutol resistance, respectively. (PDF) [file pmed.1001880.s011.pdf]

	# evolutions before genotypic resistance	# evolutions after genotypic resistance	# evolutions concurrent to genotypic resistance	# evolutions concurrent or after genotypic resistance
ahpC promoter	0	1	0	1
ubiA non-synonymous	2	6	5	11
